# Nucleolin Participates in DNA Double-Strand Break-Induced Damage Response through MDC1-Dependent Pathway

**DOI:** 10.1371/journal.pone.0049245

**Published:** 2012-11-07

**Authors:** Junya Kobayashi, Hiroko Fujimoto, Jun Sato, Ikue Hayashi, Sandeep Burma, Shinya Matsuura, David J. Chen, Kenshi Komatsu

**Affiliations:** 1 Department of Genome Repair Dynamics, Radiation Biology Center, Kyoto University, Kyoto, Japan; 2 Graduate School of Biomedical Sciences, Hiroshima University, Hiroshima, Japan; 3 Division of Molecular Radiation Biology, Department of Radiation Oncology, University of Texas Southwestern Medical Center at Dallas, Dallas, Texas, United States of America; 4 Department of Genetics and Cell Biology, Research Institute for Radiation Biology and Medicine, Hiroshima University, Hiroshima, Japan; German Cancer Research Center, Germany

## Abstract

H2AX is an important factor for chromatin remodeling to facilitate accumulation of DNA damage-related proteins at DNA double-strand break (DSB) sites. In order to further understand the role of H2AX in the DNA damage response (DDR), we attempted to identify H2AX-interacting proteins by proteomics analysis. As a result, we identified nucleolin as one of candidates. Here, we show a novel role of a major nucleolar protein, nucleolin, in DDR. Nucleolin interacted with γ-H2AX and accumulated to laser micro-irradiated DSB damage sites. Chromatin Immunoprecipitation assay also displayed the accumulation of nucleolin around DSB sites. Nucleolin-depleted cells exhibited repression of both ATM-dependent phosphorylation following exposure to γ-ray and subsequent cell cycle checkpoint activation. Furthermore, nucleolin-knockdown reduced HR and NHEJ activity and showed decrease in IR-induced chromatin accumulation of HR/NHEJ factors, agreeing with the delayed kinetics of γ-H2AX focus. Moreover, nucleolin-knockdown decreased MDC1-related events such as focus formation of 53 BP1, RNF168, phosphorylated ATM, and H2A ubiquitination. Nucleolin also showed FACT-like activity for DSB damage-induced histone eviction from chromatin. Taken together, nucleolin could promote both ATM-dependent cell cycle checkpoint and DSB repair by functioning in an MDC1-related pathway through its FACT-like function.

## Introduction

DNA double-strand breaks (DSBs) are often generated in genomic DNA upon exposure to ionizing radiation, DNA damaging agents such as bleomycin and neocarzinostatin, or due to the stalling or collapse of DNA replication forks. As unrepaired DSBs induce genome instability and promote apoptosis or tumorigenesis, cells recognize DSBs immediately, allow DNA repair factors to access DSBs and then activate DNA repair mechanisms. However, eukaryotic genomic DNA is organized into stable chromatin structures comprised of higher-order folding and condensed nucleosomes. As this stable structure impedes access of DNA-modifying factors for replication, transcription and repair, cells have evolved various mechanisms to mark and modify the chromatin landscape, including histone modifications and local recruitment of chromatin remodeling factors before activation of DNA-modifying machinery. Likewise, in order to promote access of DNA repair factors to DNA damage sites, both modifications of histones and remodeling of chromatin structure are required.

Although histones undergo different kinds of modification such as methylation, acetylation and phosphorylation, several reports suggest a tight relationship between histone acetylation and DNA damage response [1], [2]. Tamburini and Tyler showed that acetylation of histone H3 and H4 increased at HO endonuclease-generated DSB sites in yeasts, and also showed that the histone acetyltransferases Gcn5 and Esa1 were recruited to these damage sites [3]. Downs and his colleagues also reported that Arp4, a subunit of NuA4 HAT (histone acetyltransferase) complex, is recruited to HO endonuclease-induced DSB sites and interacts with phosphorylated H2A directly [4]. In addition, Ikura et al. reported that the human homolog of NuA4 HAT, Tip60, interacts with and acetylates histone H2AX, and also showed that acetylation of histone H2AX increased in response to DSB damage, and dominant-negative Tip60-expressing HeLa cells exhibit attenuated DSB repair following IR [5], [6]. Also, we reported previously that acetylation of histone H2A is indispensable for DNA damage-induced focus formation and homologous recombination (HR) repair [7]. Furthermore, chromatin remodeling factors such as INO80 and SWI/SNF appear to be important for the DNA damage response. The yeast Ino80 and Swr1 chromatin-remodeling complexes interact with the phosphorylated form of histone H2AX (γH2AX) and facilitate DSB repair [8]–[10]. Wu and his colleagues showed that knockdowns of human INO80 or a binding partner, YY1, increased cellular sensitivity toward DNA-damaging agents, and that both INO80 and YY1 are essential for HR repair [11]. Downregulation of Brg1, a component of human SWI/SNF1 complex, results in inefficient DSB repair and increased DNA damage sensitivity [12]. Thus, these and additional reports suggest that both histone modification and chromatin remodeling are important for the maintenance of genomic stability in the face of both endogenous and exogenous DNA damage.

Recently, histone ubiquitination has emerged as an important event in the DNA damage response (DDR). Histone H2A and its variant H2AX are both ubiquitinated by RNF8 and RNF168 E3 ligase, in a MDC1 (mediator of DNA damage checkpoint 1)-dependent manner, following generation of DSBs [13]–[15]. This ubiquitinated H2A/H2AX recruits HR-related factors such as RAP80, Abraxas and BRCA1, leading to activation of HR machinery [16]. MDC1 was identified as a binding partner for the hMRE11/hRAD50/NBS1 (MRN) complex and contains a forkhead-associated (FHA) domain and two BRCA1 carboxy-terminal (BRCT) domains [17]. The binding of MDC1 to γ-H2AX through its tandem BRCT domains is important for its recruitment and accumulation at DSB sites, which, in turn, facilitates activation of ATM-dependent cell cycle checkpoint and HR repair [18]. Furthermore, MDC1 is phosphorylated by ATM in response to DSB damage and this phosphorylation is responsible for the recruitment and accumulation of RNF8 and RNF168 to DSB sites [19]. These facts indicate that MDC1 is an important regulatory factor for histone H2A ubiquitination in the DNA damage response and might function in chromatin remodeling in response to DNA damage through histone ubiquitination.

H2AX is one of the variants of histone H2A and comprises 2–25% of the H2A complement in mammalian chromatin. As previously reported by us, H2AX is rapidly phosphorylated at Serine139 in an ATM-dependent manner at the sites of DSBs [20] and phosphorylated H2AX (γ-H2AX) interacts with NBS1, MDC1 and BRCA1, thereby promoting their accumulation at DSB sites [21], [22]. Hence, H2AX-deficient cells are defective in the formation of DSB-induced nuclear foci of several DNA damage-related (DDR) proteins such as NBS1 [22]–[24]. We also reported that this phosphorylation of H2AX contributes to the activation of ATM kinase [24]. Besides phosphorylation, histone H2AX is subject to other modifications such as acetylation, methylation, and ubiquitination. As mentioned above, acetylation and ubiquitination of H2A/H2AX are important for accumulation of DDR proteins and subsequent initiation of HR repair. Furthermore, γ-H2AX is also important for the recruitment of chromatin remodeling factors such as yeast Ino80 to DSB sites by direct binding through its phosphorylation site [9]. These facts suggest that H2AX is one of the key regulators of chromatin remodeling via its phosphorylation on serine139. In order to define the H2AX-dependent chromatin remodeling pathway, we attempted to identify components of the γ-H2AX complex which forms at DSB sites using GST-pull-down assays coupled with proteomics analysis. As a result, we identified nucleolin as a candidate of H2AX-interacting protein and investigated its role in DDR. Nucleolin accumulated into DSB damage sites in H2AX-dependent manner. Depletion of nucleolin by siRNA reduced accumulation of several DDR proteins and resulted in reduction of ATM-dependent cell cycle checkpoint and DSB repair. Nucleolin also seemed to be responsible for MDC1-dependent histone ubiquitination and other MDC1-related responses. Thus, nucleolin could be important for H2AX/MDC1-related DNA damage responses, and we discuss this novel function of nucleolin in DSB damage response and chromatin remodeling.

## Results

### Identification of Nucleolin as an H2AX-associated Protein

**Figure 1:**
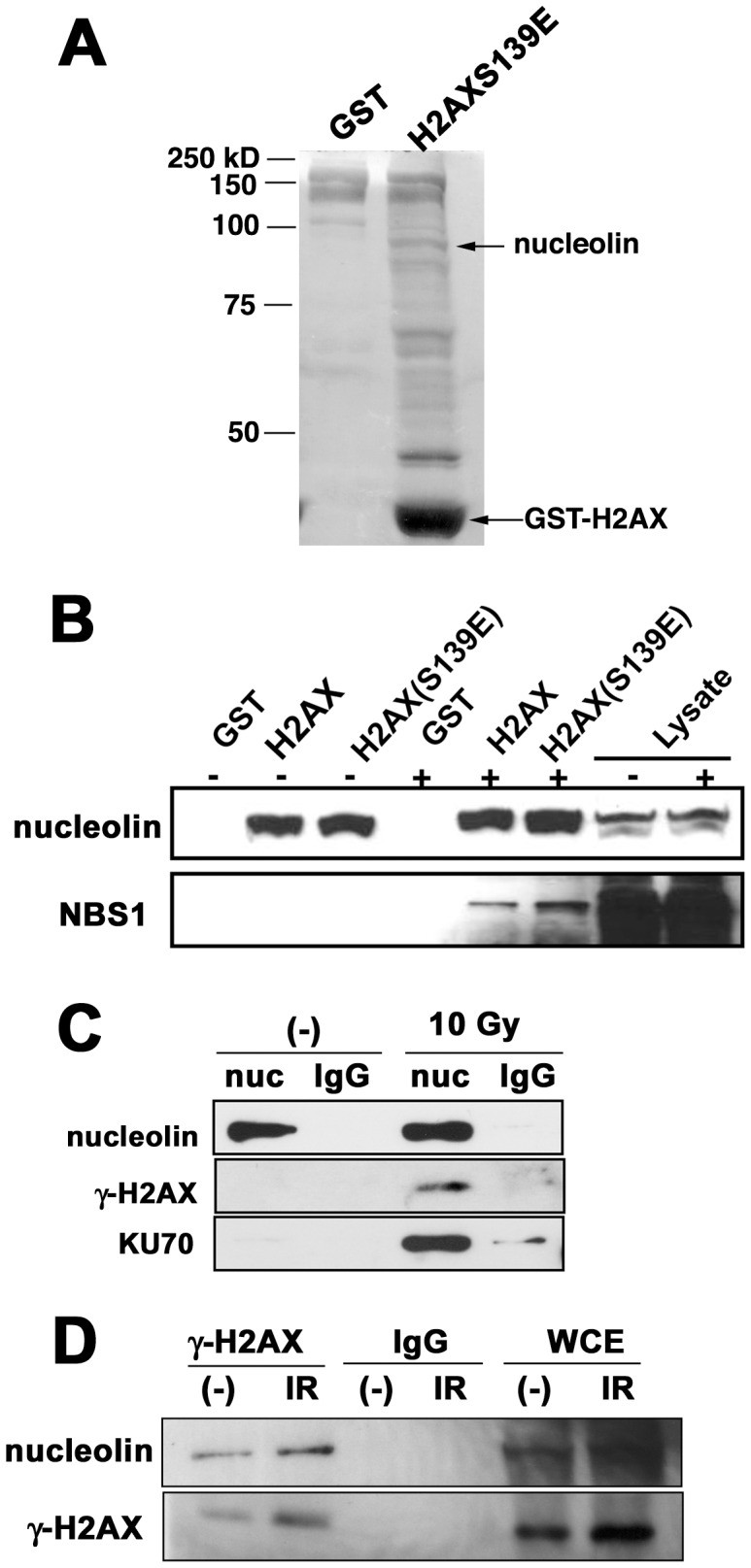
Identification of nucleolin as a γ**-H2AX-interacting protein.** (A) phospho-mimic GST-H2AX (H2AXS139E) pulls down several proteins including nucleolin from the nuclear extract of irradiated HeLa cells. Proteins were visualized by silver staining. (B) Pull-downed proteins from the nuclear extract of irradiated or un-irradiated HeLa cells were visualized by Western blot. Nucleolin was precipitated by both H2AX and H2AX (S139E), but NBS1 was precipitated by H2AX (S139E) from irradiated extract only. (C and D) Nucleolin interacts with γ-H2AX. Extracts from normal lymphoblastoid cells were immunoprecipitated with anti-nucleolin antibody (C), anti-γ-H2AX antibody (D) or normal rabbit IgG, and then the immuno-complexes were detected by Western blot analysis using indicated antibodies.

In order to identify proteins that bind to H2AX, especially phosphorylated H2AX (γ-H2AX), we designed GST-fused H2AX and GST-fused phospho-mimic H2AX, whose serine at position 139 is substituted to glutamic acid. These recombinant proteins were used to “pull down” possible associating proteins from nuclear extracts of irradiated HeLa cells. Candidate H2AX-interacting proteins were identified by peptide Mass fingerprint analysis (Fig. S1A). One of the identified proteins was the approximately 100 kDa nucleolar protein, nucleolin (Fig. 1A and S1B). Nucleolin has RNA binding activity and is important for protein translation processes [25]. Recently, it was reported that nucleolin physically interacts with Rad51 and Replication Protein A (RPA) and contributes to the tumor repressor function of p53 [26]–[28], suggesting that nucleolin may be an important player in the DSB damage response. We first confirmed whether nucleolin is actually part of the GST-H2AX-pulldown complex (Fig. 1B). Phospho-mimic H2AX (S139E) precipitated nucleolin from nuclear extracts, but unexpectedly, wild type H2AX also pulled down nucleolin. Remarkably NBS1 was also co-precipitated by phospho-mimic H2AX from nuclear extract of irradiated cells. As nucleolin has been reported to interact with histone H2A [25], we also tried GST-H2A-pulldown assay (Fig. S1DE). We noticed that the band patterns of precipitated proteins were different between GST-H2A and GST-H2A-S139E around 100 kDa (* in Fig. S1D), but Western blot analysis showed that GST-H2A precipitated similar amount of nucleolin to GST-H2AX (Fig. S1E). We next examined the endogenous interaction between nucleolin and γ-H2AX. Anti-nucleolin antibody co-precipitated γ-H2AX and the NHEJ repair factor, KU70 from irradiated cells (Fig. 1C), while another major ribosomal protein S6 did not interact with neither γ-H2AX nor Ku70 (Fig. S1C). Anti-γ-H2AX antibody also preferably precipitated nucleolin from irradiated cell extracts (Fig. 1D). As KU70/KU80 complex are rapidly recruited to DSB ends, this result indicates that nucleolin might interact with γ-H2AX at DSB sites.

### Nucleolin Accumulates at DNA Damage Sites in an H2AX-dependent Manner

**Figure 2:**
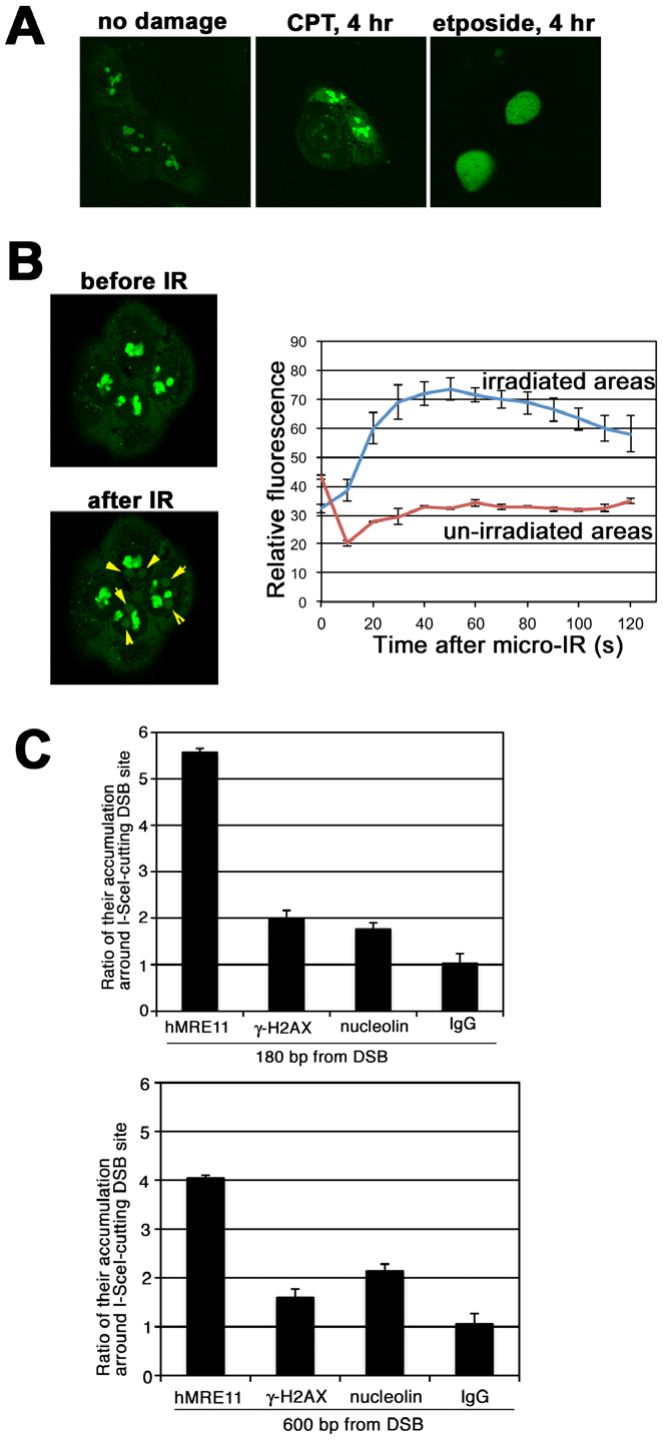
Nucleolin accumulates at DSB damage sites. (A) GFP-nucleolin is confined into nucleolus without DNA damage and diffuses to nucleolpasm after DNA-damaging treatment (CPT 1 µM; etoposide 1 µM) in HeLa cells. (B) GFP-nucleolin is rapidly recruited to DNA damage sites induced by laser micro-irradiation in U2OS cells. Accumulation of GST-nucleolin into irradiated area is indicated by yellow arrowheads. (C) Detection of nucleolin accumulation around DSB damage sites by ChIP. After transfection of I-SceI plasmids in HeLa-DRGFP cells to generate DSBs, the accumulation of γ-H2AX, nucleolin, hMRE11 around DSBs were analyzed by ChIP analysis using specific antibodies.

As a large number of DDR proteins recruited to DNA damage sites can be visualized as nuclear “foci”, we investigated the focus formation of nucleolin following generation of DNA damage (Fig. 2A). As nucleolin is a nucleolar protein, almost all GFP-nucleolin is confined to the nucleoli in the absence of DNA damage. DNA damage induction by camptothecin (CPT) and etoposide did not induce nuclear focus formation of nucleolin, but triggered its relocation from nucleoli to the nucleoplasm. We also examined the re-localization of endogenous nucleolin by immunofluorescence using anti-nucleolin antibody, but failed to observe focus formation in response to γ-rays, CPT, or etoposide (data not shown).

Although several DDR proteins, such as the NHEJ protein Ku70/80, are recruited to DSB sites, their recruitment cannot be visualized as nuclear foci, perhaps because they are localized closer to the DSB and are not spread out over megabases of chromatin as with γ-H2AX [29]. In these cases, their recruitment to DSB sites could be visualized using the laser micro-irradiation method [30], [31]. Therefore, we investigated whether nucleolin is recruited to DNA damage sites induced by laser micro-irradiation. Upon irradiation of nucleoplasm with 405 nm of laser, GFP-nucleolin relocated rapidly to the irradiated area (Fig. 2B). However, micro-irradiation of the nucleolus did not trigger relocation to irradiated area (Fig. S2A). As nucleolin was identified as an H2AX-binding protein, we examined if H2AX is required for this recruitment. H2AX (+/+) MEFs showed rapid recruitment to irradiated area, but H2AX (−/−) MEFs did not display such recruitment (Fig. S2B). Introduction of H2AX-WT to H2AX (−/−) MEFs restored the recruitment of nucleolin to irradiated area, but mutated H2AX (S139A) did not (Fig. S2C). Thus, H2AX and its phosphorylation are important for the recruitment of nucleolin to DSBs. Recently, Chromatin-immunoprecipitation (ChIP) has been extensively and effectively used to investigate the recruitment and accumulation of DDR factors to DSB sites [32]. ChIP analysis revealed that nucleolin as well as hMRE11 and γ-H2AX accumulate around DSB sites in both HeLa and MRC5SV cells (Fig. 2C and S2E). Take together, these results indicate that nucleolin can be recruited to DNA damage sites and may function in the DNA damage response.

### Nucleolin is Required for ATM-dependent Cell Cycle Checkpoint Implementation

**Figure 3:**
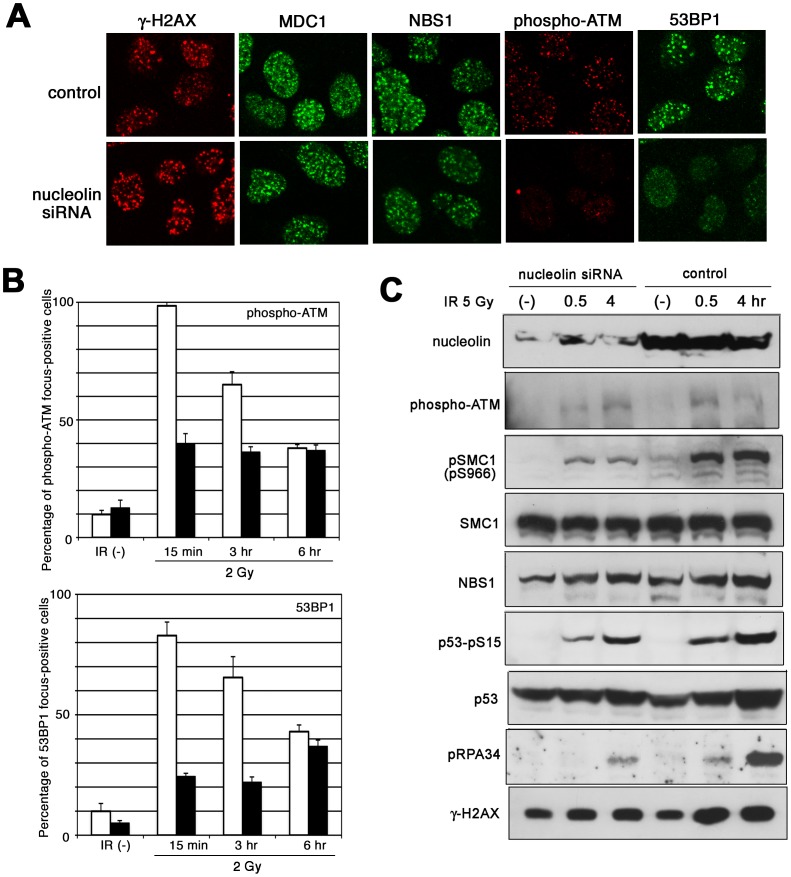
Nucleolin contributes to ATM-dependent DNA damage responses. (A and B) U2OS cells were transfected by nucleolin siRNA or negative control siRNA, and after 2 days these cells were irradiated by 5 Gy of γ-ray. After 30 minutes, their cells were fixed and immuno-staining was performed using indicated antibodies. Percentage of phospho-ATM or 53BP1 foci-positive cell at indicated times after 2 Gy of irradiation was shown in (B). Open column: control, closed column: nucleolin siRNA. (C) 48BR cells were transfected by nucelolin siRNA. After 2 days, these cells were irradiated by 5 Gy of γ-ray and were harvested at indicated times after IR and analyzed by Western blot using indicated antibodies.

In order to clarify the role of nucleolin in DDR, we designed siRNA targeting human nucleolin, which effectively reduced the expression of nucleolin protein (Fig. S3A). Although we identified nucleolin as an H2AX-interacting protein, U2OS cells with knockdown of nucleolin showed normal γ-H2AX foci formation after irradiation (Fig. 3A and S3B). MDC1, NBS1 and MRE11 also showed normal focus formation following irradiation. Very interestingly, focus formation by phosphorylated-ATM was reduced in nucleolin-knockdown U2OS cells (Fig. 3AB and S3C), and similar results were observed in HeLa cells (Fig. S3D). As auto-phosphorylation of ATM is known to regulate its kinase activity and subsequent cell cycle checkpoints, we interrogated the nucleolin-depleted cells for defects in ATM-mediated DNA damage responses. Nucleolin-depleted 48BR and MRC5SV cells exhibited reduced ATM auto-phosphorylation and reduced phosphorylation of its substrates SMC1, NBS1, p53, Chk2, RPA34, DNA-PKcs and γ-H2AX, but depletion of nucleolin did not influence the expression of their proteins (Fig. 3C and S4AB). A different nucleolin siRNA (nucleolin siRNA2) also remarkably reduced nucleolin and repressed the phosphorylation of ATM and SMC1 (Fig. S4C), suggesting that the results in Figures 3C and S4A are not due to off-targets effects of the siRNA being used. Furthermore, nucleolin-knockdown abrogated G2 checkpoint after irradiation, although accumulation in G2 phase was observed 12 hours after irradiation of control cells with 10 Gy of IR (Fig. S4D). Furthermore, formation of 53BP1 foci following IR was also repressed in nucleolin-knockdown cells (Fig. 3AB and S3CD), although ATM-deficient AT cells can still form 53BP1 foci in response to IR [33]. Probably, nucleolin could participate in ATM-dependent DSB damage responses and independent-DSB responses such as 53BP1 focus formation.

### Deficiency in DNA Damage Response Upon Nucleolin Knockdown is not Due to its Nucleolar Function

**Figure 4:**
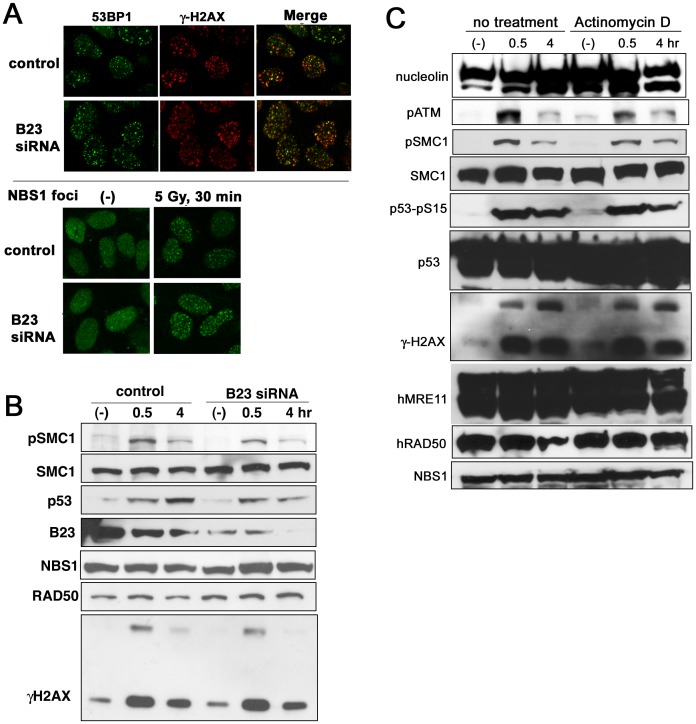
Nucleolus-related function of nucleolin might be dispensable for of its role in the DNA damage response. (A and B) U2OS cells were transfected by nucleophosmin (B23) siRNA, and after 2 days these cells were irradiated by 5 Gy of γ-ray. After 30 minutes, their cells were fixed for immuno-staining (A), while their cells were harvested at indicated time for Western blot analysis (B). (C) U2OS cells were irradiated by 5 Gy of γ-ray with or without pre-treatment of actinomycin D (0.02 mg/ml, 1 hour). These cells were harvested at indicated times after IR and analyzed by Western blot using indicated antibodies.

Nucleolin forms a complex with nucleophosmin and this complex functions in nucleolus formation and ribosomal RNA (rRNA) synthesis [25]. Besides, recent reports suggest that nucleophosmin (B23) is important for translocation of p19ARF in response to DNA damage [34]. Hence, we investigated whether repression of nucleophosmin by siRNA causes effects similar to nucleolin knockdown. siRNA-mediated depletion of nucleophosmin did not affect the formation of 53BP1 foci after irradiation (Fig. 4A), contrary to the abrogation of 53BP1 foci seen after nucleolin knockdown (Fig. 3AB). ATM-dependent phosphorylation was also not influenced by nucleophosmin knockdown (Fig. 4B), suggesting that the defective DNA damage responses in nucleolin-depleted cells are independent of its binding partner, nucleophosmin. As nucleolin is also important for rRNA synthesis, we also evaluated whether a deficiency in rRNA synthesis might abrogate ATM-related DNA damage responses (Fig. 4C). Treatment of mammalian cells with low concentrations of actinomycin D (0.02 mg/ml) repressed rRNA synthesis [35], but this treatment did not change the phosphorylations of ATM, SMC1, p53 and H2AX (Fig. 4C). Taken together, nucleolin could play a role in the DNA damage response due to a specific novel function, which is distinct from its role in nucleolus formation and rRNA synthesis.

### Nucleolin Could Participate in DNA Double-strand Break Repair

**Figure 5:**
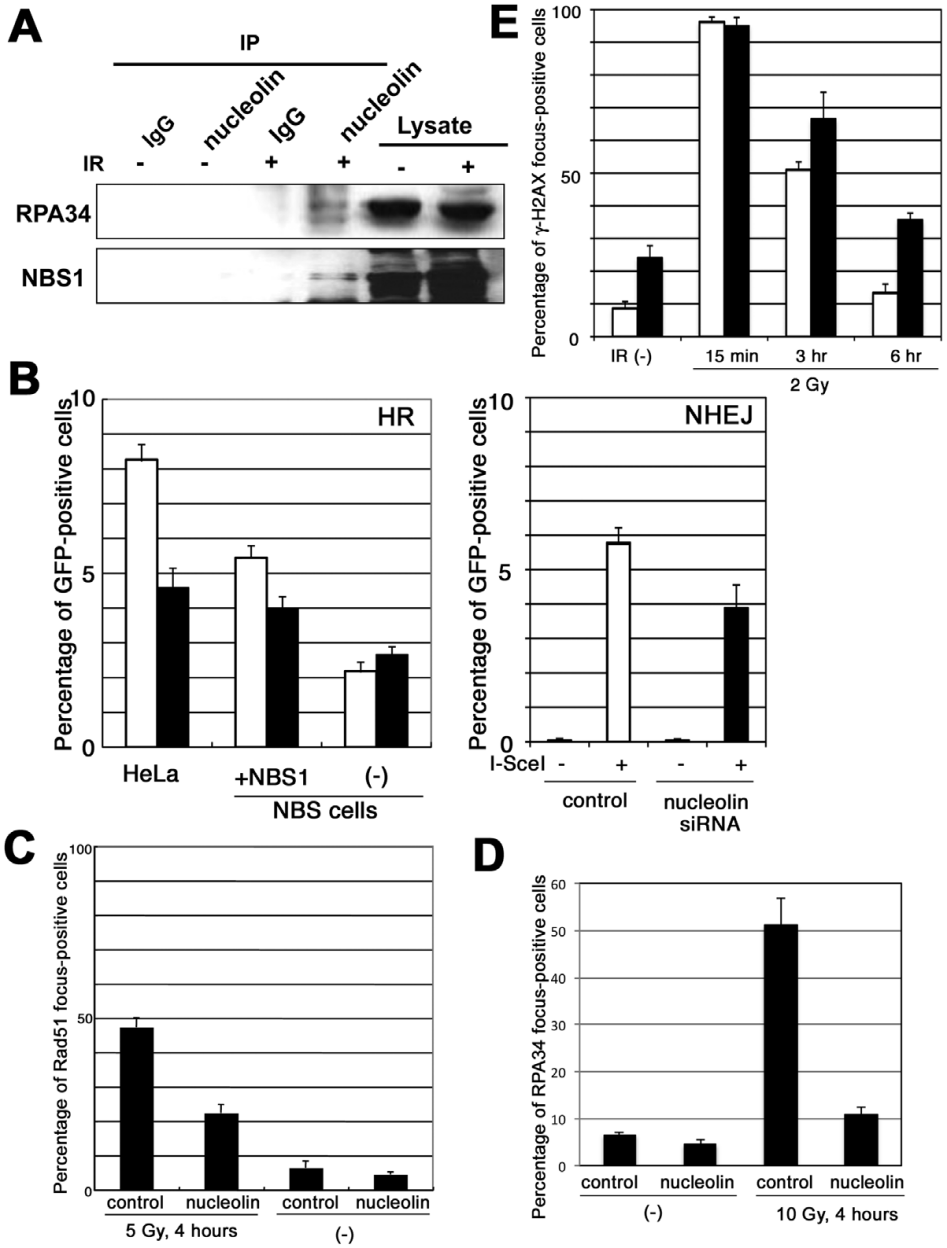
Nucleolin participates in DSB repair. (A) Nucleolin interacts with HR factors, RPA34 and NBS1 in response to DNA damage. The extracts from normal lymphoblastoid cells were immunoprecipitated with anti-nucleolin antibody or normal rabbit IgG, and then the immuno-complexes were detected by Western blot analysis. (B) NHEJ and HR activity in nucleolin-knockdown cells. HeLa-DRGFP, NBS-DRGFP or MRC5-pEJ cells were transfected by nucleolin siRNA. After 2 days I-SceI expression plasmid were introduced to these cells by electroporation and analyzed by flowcytometer. Open column: control, closed column: nucleolin siRNA. (C, D and E) U2OS cells were transfected by nucleolin siRNA or negative control siRNA, and after 2 days these cells were irradiated by 5 Gy of γ-ray. After indicated times, their cells were fixed and immuno-staining was performed using anti-Rad51 antibody (C), anti-RPA 34 antibody (D) or anti-γ-H2AX antibody (E). Then, percentages of foci-positive cell were counted under fluorescence microscope. Open column: control, closed column: nucleolin siRNA.

DNA double-strand break repair is very important for cell survival after the induction of DSBs. DSB repair involves two major pathways, NHEJ and HR. In order to elucidate the role of nucleolin in DSB repair, we first examined the interaction of nucleolin with DSB repair factors. Fig. 5A shows that anti-nucleolin antibody co-precipitated important HR factors, RPA34 and NBS1, suggesting a role of nucleolin in the HR pathway. We also estimated the importance of nucleolin in HR activity using a GFP-based HR reporter system [36] after siRNA-mediated knockdown of nucleolin (Fig. 5B). The generation of DSB by expression of I-SceI restriction enzyme induced approximate 8% of GFP-positive cells *via* HR repair pathway, but nucleolin repression by siRNA decreased this activity by about half. As NBS1 is important factor for HR repair, NBS1-deficient NBS cells also show a low frequency of GFP-positive cells (approximate 2%; Fig. 5B). Repression of nucleolin in NBS cells did not further decrease this HR frequency, suggesting that nucleolin could function for HR repair in an NBS1-related pathway. Importantly, IR-induced focus formation of HR factors such as Rad51 and BRCA1 was also reduced in nucleolin-depleted cells (Fig. 5C and S5A). Resection of DSB ends is one of critical initial reaction for HR repair pathway, and this resection reaction could be visualized by RPA34 focus. When we observed the focus formation of RPA34 following irradiation, formation of RPA34 focus was repressed in nucleolin-depleted cells (Figure 5D and S5C). Reduction of RPA34 phosphorylation in nucleolin-depleted cells (Fig. 3C) also supports the role of nucleolin in HR repair. Although nucleolin is also important for transcription and translation, the repression of nucleolin did not change the expressions of HR factors (Fig. S5B), indicating that nucleolin could participate in HR machinery directly, perhaps by facilitating the initial resection and subsequent recruitment/accumulation of key HR proteins such as RPA, Rad51 and BRCA1 at DSB sites.

As nucleolin interacts with KU70 (Fig. 1C), we also examined NHEJ activity using a GFP-based NHEJ reporter system [37]. Fig. 5B shows that the generation of DSB by I-SceI induced approximately 6% of GFP-positive cells *via* NHEJ repair pathway, but the repression of nucleolin reduced this activity, suggesting that nucleolin might also contribute to NHEJ repair partially. As these results suggest the contribution of nucleolin to both HR and NHEJ repair pathways, we estimated the deficiency of DSB repair in nucleolin-deficient cells using γ-H2AX foci as a surrogate marker for DSBs (Fig. 5E and S5D). At 15 minutes after exposure to 2 Gy of γ-ray, more than 90% of both control and the deficient cells showed γ-H2AX foci, but after 6 hours, most control cells exhibited diminished γ-H2AX foci. However, nucleolin-deficient cells retained γ-H2AX foci after 6 hours, indicating attenuated DSB repair. Similar results were obtained when NBS1 foci were used to quantify DSBs (Fig. S5E). Taken together, these results indicate that nucleolin is important for DNA double-strand break repair.

### Nucleolin is Required for MDC1-dependent Chromatin Modification and Histone Eviction

**Figure 6:**
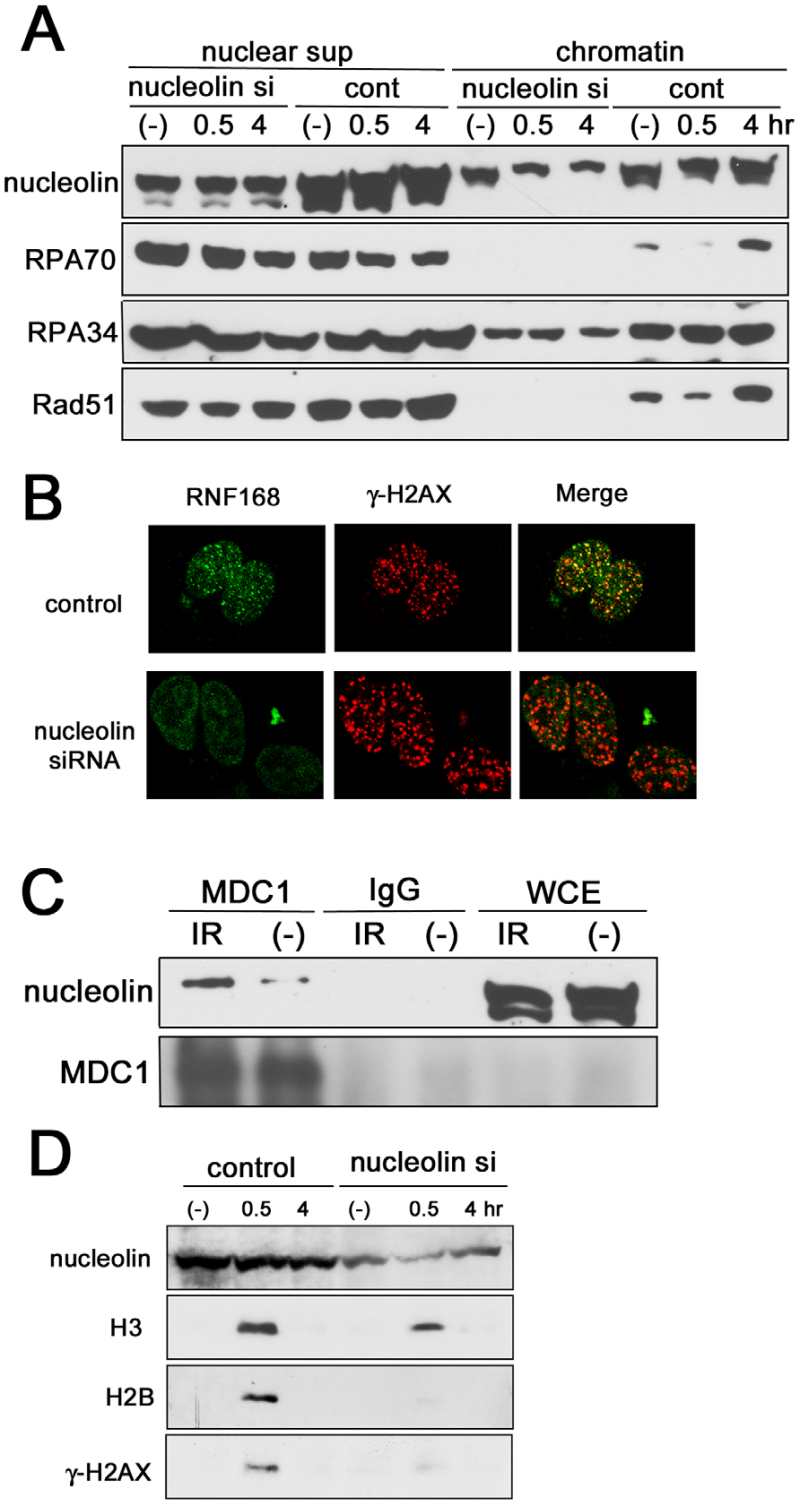
Nucleolin contributes to MDC1-dependent damage responses and histone eviction. (A) Nucleolin is required for IR-induced accumulation of HR factors into chromatin fraction. U2OS cells were transfected by nucleolin siRNA. After 2 days, these cells were irradiated by 10 Gy of γ-ray and were harvested at indicated times after IR. Nucleoplasm (nuclear sup) and chromatin extracts were prepared as described in Materials and Methods, and analyzed by Western blot using indicated antibodies. (B) Nucleolin is required for RNF168 focus formation. U2OS cells were transfected by nucleolin siRNA or negative control siRNA, and after 2 days these cells were irradiated by 5 Gy of γ-ray. After 30 minutes, their cells were fixed and immuno-staining was performed using indicated antibodies. (C) Nucleolin interacts with MDC1. Extracts from normal lymphoblastoid cells were immunoprecipitated with anti-MDC1 antibody or normal rabbit IgG, and then the immuno-complexes were detected by Western blot analysis using anti-nucleolin antibody. (D) IR-induced histone release to nucleoplasm was abolished by depletion of nucleolin. U2OS cells were transfected by nucleolin siRNA. After 2 days, these cells were irradiated by 10 Gy of γ-ray and were harvested at indicated times after IR. After preparation of nucleoplasm (nuclear sup) and chromatin, histone proteins in nucleoplasm were analyzed by Western blot.

As most of DDR factors are recruited to DNA damage sites *via* their association with chromatin, we examined the chromatin association of key DDR proteins following irradiation and the effect of nucleolin knockdown on the process. HR factors, Rad51, RPA34 and RPA70 in control cells accumulated into chromatin fraction at 4 hours after 5 Gy of irradiation, while they did not show chromatin association without DNA damage (Fig. 6A). However, nucleolin-depleted cells did not accumulate Rad51 and RPA with or without DNA damage. ATM and the NHEJ factor, KU80 also showed a similar deficiency (Fig. S6A). They accumulated into chromatin fraction in control cells after irradiation, but depletion of nucleolin abrogated these accumulations, in agreement with the deficiency of DNA-PK auto-phosphorylation and ATM-dependent phosphorylation in knockdown cells (Fig. 3C and S4A). Recent reports have shown that ubiquitination of histone H2A/H2AX is important for DSB damage response, particularly HR repair pathway [19]. Hence, we examined H2A ubiquitination-related pathway in nucleolin-depleted cells. Nucleolin knockdown decreased both ubiquitination of H2A and γ-H2AX and focus formation of RNF168 E3 ligase (Fig. 6B and S6C), which is responsible for H2A/H2AX poly-ubiquitination. MDC1 is important for accumulation of RNF8/RNF168 to DNA damage sites, but IR-induced chromatin association of MDC1 was also reduced (Fig. S6C). Furthermore, Fig. 6C shows the physical interaction of nucleolin with MDC1. These results suggest that nucleolin is important for MDC1-dependent H2A/H2AX ubiquitination pathway. H2B ubiquitination was also reduced in the knockdown cells (Fig. S6C), although this ubiquitination is independent of MDC1-related pathway [38].

Recently, it was reported that nucleolin is required for eviction of histone H2A/H2B complex from nucleosome through its FACT (facilitates chromatin transcription)-like activity at transcriptionally active sites [39]. We speculated that nucleolin is also important for histone eviction following DNA damage. Control cells showed the release of histone H2B, H3 and γ-H2AX to nucleoplasm from chromatin, but nucleolin-depleted cells exhibited remarkably decreased these releases (Fig. 6D). However, nucleolin knockdown did not influence the expression of these histones (Fig. S6D). Taken together, nucleolin could be important for histone eviction following DNA damage and this eviction response may be important for MDC1-dependent H2A/H2AX ubiquitination pathway.

## Discussion

We report here that nucleolin participates in the DNA double-strand break-induced DNA damage response, particularly *via* MDC1-dependent pathway. We indentify nucleolin as an H2AX-interacting protein and show that nucleolin can accumulate at DSB damage sites by laser micro-irradiation and ChIP analysis (Fig. 2 and S2). Although nucleolin might not be important for focus formation of early factors such as γ-H2AX, NBS1/MRE11 complex and MDC1 (Fig. 3A), nucleolin could be involved in ATM-dependent phosphorylation of its substrates (Fig. 3C and S4AC). Furthermore, nucleolin-knockdown cells exhibited delayed kinetics of resolution of IR-induced γ-H2AX foci indicating a deficiency in DSB repair (Fig. 5E and S5D), especially due to a defect in the HR pathway of repair (Fig. 5BC and S5A). Repression of nucleolin also decreased focus formation of 53BP1, BRCA1 and RNF168, and ubiquitination of H2A/H2AX (Fig. 3AB, 6B, S3CD and S6C). Several reports indicated that focus formation of 53BP1, BRCA1 and RNF168 are dependent on ubiquitination of H2A/H2AX in response to DNA damage [19]. RNF8 and RNF168 E3 ligases are responsible for H2A/H2AX mono- and poly-ubiquitination and these IR-induced accumulations at DSB damage sites require MDC1 [13]–[15]. MDC1 is also important for amplification of the ATM-related pathway [18], while repression of nucleolin reduced phosphorylated-ATM focus and ATM-related responses (Fig. 3, S3CD and S4AC). Moreover, MDC1 formed a complex with nucleolin dependently on DSB damage (Fig. 6C). Therefore, nucleolin could be responsible for MDC1-related DNA damage responses. This thought is in agreement with results showing that DNA damage-induced accumulation of MDC1 to chromatin was abolished in nucleolin-knockdown cells (Fig. S6C), although MDC1 focus formation was unchanged in their cells (Fig. 3A). Nucleolin, which is known to possess FACT-like activity through an eviction of histone H2A/H2B complex from nucleosome at transcriptionally active sites, was also indispensable for DNA damage-induced eviction of histones (Figure 6D). Figure S2B suggests that nucleolin could recruit to DSB sites in H2AX-dependent manner. Therefore, it is suggested that histone (H2A/H2B) eviction by nucleolin, following its H2AX-dependent recruitment to DNA damage sites, could facilitate MDC1-relared DNA damage response including HR repair and ATM-mediating cell cycle checkpoint (Figure S7).

Nucleolin is a major nucleolar protein containing RNA-binding motifs in the central region. Nucleolin is required for rRNA processing in nucleolus and has many other diverse functions such as transcription regulation, modulation of mRNA stability and DNA replication and recombination [25]. Recently, it was reported that nucleolin possesses FACT-like activities, stimulating SWI/SNF-mediated transfer of H2A/H2B in vitro and facilitating transcription in vitro just like FACT [39]. Hence, we hypothesize that this activity of histone transfer may be important for the DNA damage response. Several reports indicate that DSB damage stimulates histone loss around DSB damage sites in both mammalian cells and yeast using ChIP assay, and that this histone loss is followed by DSB repair response [32], [40]. Indeed, Fig. 6D shows that histones were released from chromatin in response to DSB damage, but depletion of nucleolin abolished these releases. Thus, histone transfer/loss from chromatin around DNA damage sites might be important for the nucleolin-dependent regulation of DNA damage response and this nucleolin-dependent histone transfer/loss may be important for stimulation of MDC1-dependent DSB signaling pathway. It was also reported that nucleolin physically interacted with HR factors such as Rad51 and RPA [26], [27]. Hence, these interactions may contribute to nucleolin-dependent regulation of HR independently of histone release.

It has been suggested that histone modification and subsequent chromatin remodeling is closely linked with DSB repair, particularly HR repair. We and other groups reported that Tip60-dependent histone acetylation is important for the HR repair pathway [7], [41]. Our report showed that repression of Tip60-dependent acetylation of histone H2A/H2AX reduced focus formation of HR-related factors such as NBS1 and MRE11, and led to decreases in HR activity [7]. Murr et al. reported that a binding partner of Tip60, TRAPP-knockout mouse cells showed a decrease in histone acetylation around DSB sites, IR-induced focus formation of HR factors and HR activity [41]. Tsukuda and her colleagues reported that INO80-dependent chromatin remodeling regulated early and late stage of mitotic homologous recombination [40]. These facts indicate the importance of histone modification and chromatin remodeling for HR repair, but their relationships with NHEJ have been unclear. Recently, Ogiwara *et al.* showed that CBP and p300-dependent histone acetylation could facilitate the recruitment of KU proteins and NHEJ repair activity [42]. Moreover, the chromatin remodeling factor, ACF1 was suggested to participate in NHEJ repair through initial recruitment of KU proteins to DSB sites [43]. These reports suggest that histone modification and subsequent chromatin remodeling might also contribute to the control of NHEJ repair. In fact, our data showed that nucleolin interacts with KU70 in response to DNA damage (Fig. 1C) and that nucleolin knockdown reduced both auto-phosphorylation of DNA-PKcs and NHEJ activity (Fig. S4A and 5B). Therefore, nucleolin-dependent chromatin remodeling might also be important for NHEJ pathway. As nucleolin contributes to ACF1-mediated nucleosome sliding activity [39], nucleolin may participate in NHEJ through the function of ACF1 to nucleosome.

As described above, nucleolin is one of the most abundant non-ribosomal proteins of the nucleolus and is important for nucleolar formation and rRNA metabolism. Hence, we investigated the possibility that the defect in DNA damage responses for depletion by siRNA is due to the deficiency of normal nucleoli formation in those cells. However, both knockdown of nucleophosmin, a binding partner for nucleolin, and repression of rRNA synthesis by actinomycin D did not show any defect in the DNA damage response (Fig. 4). These results suggest that the function of nucleolin in the DNA damage response is distinct from its role in the nucleolus. It is known that several DNA repair-related proteins such as WRN, BLM, RECQL4, and aprataxin mainly localizes in the nucleolus and many DNA repair proteins are present in the nucleolus fraction [44]. Nucleolus contains high copies of ribosomal RNA gene (rDNA) and the maintenance of this copy number is important for genome integrity in yeast [45], [46]. Moreover, amplification of rDNA requires HR-related proteins such as RAD52 and MRE11 [47]. These reports suggest the importance of HR pathway for rDNA maintenance and the existence of nucleolus-specific DNA repair mechanisms. In fact, it was reported in yeast that the Smc5-Smc6 complex and sumoylated Rad52 regulate recombination repair at the ribosomal gene locus, and that the maintenance of rDNA could be performed by perinuclear chromosome tethering system [47]. Hence, mammalian cells may also have a specific system for rDNA maintenance using HR. As our results show here that nucleolin could participate in HR repair for genome integrity, nucleolin may also contribute to the repair system for rDNA. Several observations suggest that nucleolin is up-regulated in human tumors and overexpression of nucleolin cooperates with oncogenic mutated Ras in transformation of rat embryonic fibroblast and human cancers [28], [48]. Therefore, it will be important to further clarify the role of nucleolin in genome integrity and rDNA maintenance.

## Materials and Methods

### Cell Culture

HeLa, U2OS, hTERT-immortalized human fibroblast 48BR [49], were cultured in DMEM (Sigma) supplemented with 10% FBS (Invitrogen) and antibiotics. H2ax −/− and H2ax +/− mouse fibroblasts were supplied by Dr. A. Nussenzweig and were cultured under above condition. Normal human lymphoblastoid GM2184 [21] were cultured in RPMI (Sigma) supplemented with 10% FBS (Invitrogen) and antibiotics.

### Antibodies

Phospho-ATM (S1981) mouse monoclonal and γ-H2AX mouse monoclonal antibodies (Millipore Co.), phospho-SMC1 (S966) rabbit polyclonal and SMC1 rabbit polyclonal, MDC1 rabbit polyclonal and Phospho RPA32 (S4/S8) rabbit polyclonal antibodies (Bethyl Laboratories Inc.), phospho-p53 (S15) mouse monoclonal, phospho-Chk2 (T68) rabbit polyclonal antibodies (Cell Signaling Technology), hMre11 rabbit polyclonal and Nbs1 rabbit polyclonal antibodies (Novus Biologicals), and nucleolin rabbit polyclonal and mouse monoclonal and p53 mouse monoclonal antibodies (Santa Cruz Biotechnology), and 53BP1 rabbit polyclonal, BRCA1 mouse monoclonal and Rad51 rabbit polyclonal antibodies (Merk Co.) and anti-RPA antibody (for RPA34; Calbiochem) and anti-Ubiquitil-Histone H2A (Upstate) were used for Western blot analysis or immuno-staining. Anti-RNF168 antibody was supplied by Dr. D. Durocher.

### SiRNA Knockdown Experiments

Sub-confluent cells, seeded culture dishes the day before, were transfected with nucleolin siRNA (Be-Bridge International Inc. and QIAGEN Co.) nucleophosmin siRNA (Santa Cruz Co.), or negative control siRNA (Be-Bridge International Inc.) using lipofectamine 2000 (Invitrogen Life Technology). After 2 days, these cells were re-seeded in proper culture dishes. Next day, these cells were used for Western blot or immunofluorescence, HR and NHEJ analysis.

### GST-pull-down and Mass Spectrometry Analysis

GST-fused human H2AX (wild type) was generated by insertion of PCR product of human H2AX cDNA into pGEX-2T (GE healthcare CO). GST-fused mutated H2AX (S139E) was generated by PCR using primers containing mutations within the codon for serine 139. Purified GST-fused proteins were used for pull-down of interacting proteins from HeLa nuclear extracts as previously reported [21]. These candidate proteins were identified using general peptide mass fingerprint analysis by autoFLEX (Bruker Dartonics).

### Laser Micro-irradiation

Cells were transfected with EGFP-nucleolin plasmid, supplied by Dr. J.A. Borowiec (26). After two days, laser micro-irradiation analysis was performed as previously described (24) using a con-focal laser microscopy (TCS-SP5; Leica Co.).

### Chromatin Immunoprecipitation (ChIP) Assay

To generate DSBs by I-SceI restriction enzyme, 1×10^6^ HeLa-DRGFP cells were pre-treated with 10 mM of Nu7026 (DNA-PKcs inhibitor; Calbiochem) for 1 hour and then, 50 µg of the I-SceI expression vector pCBASce was introduced to them by electroporation (BIO-RAD). At 1 day after transfection, cells on dishes were treated with 1% formaldehyde at 37 C for 10 min followed by the addition of 0.125 M glycine, after that their cells were harvested by a scraper. Chromatin Immunoprecipitation assay using their harvested cells was performed previously reported [38]. The precipitated DNA were then dissolved in 50 ml of TE and analyzed by quantitative real-time PCR with the ABI PRISM7500 system using Power SYBR Green PCR Master Mix (Applied Biosystems). Primers used for detection of the *I-SceI* break site were Sce180-F (5′-CATGCCCGAAGGCTACGT) and Sce180-R (5′-CGGCGCGGGTCTTGTA). As an internal control for normalization of the specific fragments amplified, human GAPDH locus was amplified using whole genomic DNA with GAPDH-F (5′-TCTCCCCACACACATGCACTT) and GAPDH-R (5′-CCTAGTCCCAGGGCTTTGATT).

### HR and NHEJ Analysis

HR and NHEJ analysis was performed as previously reported [7]. To measure the HR or NHEJ repair of I-SceI-generated DSBs, 50 µg of the I-SceI expression vector (pCBASce) was introduced to 1000000 Hela-DRGFP (for HR), NBS-DRGFP (for HR) or MRC5SV-pEJ (for NHEJ) cells cells, by electroporation (GenePulser; BIO-RAD). To determine the amount of HR or NHEJ repair, the percentage of GFP-positive cells was quantified by flow cytometer at 3 days after electroporation with FACAcalibur (Becton Dickinson).

### Preparation of Chromatin Fraction

The cell lysates with or without DNA damaging treatment were fractionated into cytoplasmic solution and nuclei. Separated nuclei was resuspended into 2 × RIPA buffer (DNA damage-treated or untreated cells were lysed in IP buffer (300 mM sodium chloride, 2 mM EDTA, 20 mM Tris/HCl at pH7.5, 0.1% Sodium deoxycholate, 0.2% SDS and 0.2% NP40) containing a protease inhibitor cocktail (Roch) and sodium orthovanadate for 15 min. Lysates (nucleoplasm fraction) were centrifuged at 20,000 ×g for 30 min to remove un-soluble debris.), and remaining nuclear pellet was resuspended in SDS sample buffer and incubated at 95°C to release the chromatin-bound proteins (chromatin fraction) for Western blot analysis.

## Supporting Information

Figure S1 **Identification of nucleolin as an associating protein with** γ**-H2AX.** (A) List of H2AX-binding protein candidates by proteomics analysis. (B) The result of MASCOT analysis about the band identified as nucleolin. (C) Extracts from normal lymphoblastoid cells with or without IR (10 Gy) were immunoprecipitated with anti-ribosomal protein S6 antibody or normal rabbit IgG, and then the immuno-complexes were detected by Western blot analysis using indicated antibodies. (D)(E) Pulldowns by GST-H2A or GST H2AX were carried out from the nuclear extract of HeLa cells. Proteins were visualized by CBB staining (D). Precipitated nucleolin was visualized by Western blot (E). Extract: input nuclear extract only.
(TIF)

Figure S2 **Nucleolin accumulates to DSB damage sites.** (A) GFP-nucleolin did not accumulate in nucleolus following laser micro-irradiation in U2OS cells. (B) Laser micro-irradiation was performed in H2AX (+/+) or H2AX (−/−) mouse cells. Green line: fluorescence at micro-irradiated area, Purple line: fluorescence at un-irradiated sites. (C) H2AX (−/−) mouse cells were transfected by GFP-nucleolin and FLAG-H2AX (WT) or FLAG-H2AX (S139A), and after 2 days laser micro-irradiation was performed. (D) Expression of ectopic H2AX and its phosphorylation in (C) were confirmed by Western blot using anti-FLAG antibody and anti-γ-H2AX antibody. FLAG-H2AX (S139A)-expressing cells also showed its phosphorylation, suggesting that other SQ motifs such as serine 135) in H2AX may be phosphorylated in response to DSB damage. (E) Detection of nucleolin accumulation around DSB damage sites in MRC5SV by ChIP analysis.
(TIF)

Figure S3 **IR-induced focus formation of nucleolin-knockdown cells.** (A) Our designing siRNA effectively reduced nucleolin protein in HeLa cells. (B)(C) U2OS cells were transfected by nucleolin siRNA or negative control siRNA, and after 2 days these cells were irradiated by 5 Gy of γ-ray. After 30 minutes, their cells were fixed and immuno-staining was performed using anti-MRE11 antibody (B) or indicated antibodies (C). phospho-ATM (red) or 53 BP1 (green) foci-positive cell were counted and these data are shown in Figure 3B. (D) Nucleolin-knockdown repressed the focus formation of phospho-ATM and 53 BP1. HeLa cells were transfected by nucleolin siRNA or negative control siRNA, and after 2 days these cells were irradiated by 5 Gy of γ-ray. After 30 minutes, their cells were fixed and immuno-staining was performed using indicated antibodies.
(TIF)

Figure S4 **Nucleolin contributes to ATM-related pathway.** MRC5SV cells (A) were transfected by nucleolin siRNA, while U2OS cells were transfected by nucleolin siRNA (B) or nucleolin siRNA2 (QIAGEN)(C). After 2 days, these cells were treated by 5 Gy of γ-ray and were harvested at indicated times after treatment, and analyzed by Western blot using indicated antibodies. (D) Nucleolin-knockdown abolished G2 checkpoint. 48BR cells were transfected by nucleolin siRNA. After 2 days, these cells were irradiated by 10 Gy of γ-ray and were fixed at indicated times by ethanol. After staining them by propidium iodide, the distribution of cell cycle was analyzed by flowcytometer. Blue column, G1 phase; red column, S phase; yellow column, G2/M phase cells.
(TIF)

Figure S5 **Nucleolin participates in DSB repair pathway.** U2OS cells were transfected by nucleolin siRNA or negative control siRNA, and after 2 days these cells were irradiated by γ-ray. Their cells were fixed and immuno-staining was performed using anti-Rad51 and anti-BRCA1 (A), anti-RPA34(C), anti-γ-H2AX (D) or anti-NBS1 (E) antibodies. Percentage of focus-positive cells at indicated times after irradiation were counted under fluorescence microscope. Open column: control, closed column: nucleolin siRNA. (B) 48BR cells were transfected by nucleolin siRNA. After 2 days, these cells were irradiated by 5 Gy of γ-ray and were harvested at indicated times after IR and analyzed by Western blot using indicated antibodies.
(TIF)

Figure S6 **Nucleolin contributes to MDC1-dependent damage responses.** (A) IR-induced accumulation of KU and ATM was abolished by repression of nucleolin. U2OS cells were transfected by nucleolin siRNA. After 2 days, these cells were irradiated by 10 Gy of γ-ray and were harvested at indicated times after IR. After preparation of nucleoplasm (nuclear supernatant) and chromatin extracts, chromatin association of KU86 and ATM was analyzed by Western blot. (B) U2OS cells were transfected by nucleolin siRNA or negative control siRNA, and after 2 days these cells (without irradiation) were immuno-stained using anti-RNF168 antibody. (C)(D) U2OS cells were transfected by nucleolin siRNA. After 2 days, these cells were irradiated by 10 Gy of γ-ray and were harvested at indicated times after IR. After preparation of chromatin extracts, chromatin associated proteins were analyzed by Western blot using indicated antibodies. Ubiquitination of H2AX was estimated with its molecular weight using anti-γ-H2AX antibody.
(TIF)

Figure S7 **Nucleolin participates into MDC1-related DNA damage responses through histone eviction.** Nucleolin recruits to DSB damage sites in H2AX-dependent manner and then promotes histone eviction and subsequent histone remodeling through binding with histone H2A/H2B. This histone eviction and remodeling facilitates chromatin association of MDC1 at DSB sites. As a result, MDC1-related DNA damage responses, such as ATM-dependent checkpoint and HR repair, are initiated.
(TIF)

## References

[1] van Attikum H, Gasser SM (2009) Crosstalk between histone modifications during the DNA damage response. Trends Cell Biol 19: 207–217. 19342239 10.1016/j.tcb.2009.03.001

[2] Xu Y, Price BD (2011) Chromatin dynamics and the repair of DNA double strand breaks. Cell Cycle 10: 261–267. 21212734 10.4161/cc.10.2.14543 PMC3048797

[3] Tamburini BA, Tyler JK (2005) Localized histone acetylation and deacetylation triggered by the homologous recombination pathway of double-strand DNA repair. Mol Cell Biol 25: 4903–4913. 15923609 10.1128/MCB.25.12.4903-4913.2005 PMC1140608

[4] Downs JA, Allard S, Jobin-Robitaille O, Javaheri A, Auger A, et al (2004) Binding of chromatin-modifying activities to phosphorylated histone H2A at DNA damage sites. Mol Cell 16: 979–990. 15610740 10.1016/j.molcel.2004.12.003

[5] Ikura T, Tashiro S, Kakino A, Shima H, Jacob N, et al (2007) DNA damage-dependent acetylation and ubiquitination of H2AX enhances chromatin dynamics. Mol Cell Biol 27: 7028–7040. 17709392 10.1128/MCB.00579-07 PMC2168918

[6] Ikura T, Ogryzko VV, Grigoriev M, Groisman R, Wang J, et al (2000) Involvement of the TIP60 histone acetylase complex in DNA repair and apoptosis. Cell 102: 463–473. 10966108 10.1016/s0092-8674(00)00051-9

[7] Kobayashi J, Kato A, Ota Y, Ohba R, Komatsu K (2010) Bisbenzamidine derivative, pentamidine represses DNA damage response through inhibition of histone H2A acetylation. Mol Cancer 9: e34. 10.1186/1476-4598-9-34 PMC2831819 20144237

[8] Park JH, Park EJ, Hur SK, Kim S, Kwon J (2009) Mammalian SWI/SNF chromatin remodeling complexes are required to prevent apoptosis after DNA damage. DNA Repair 8: 29–39. 18822392 10.1016/j.dnarep.2008.08.011

[9] van Attikum H, Fritsch O, Hohn B, Gasser SM (2004) Recruitment of the INO80 complex by H2A phosphorylation links ATP-dependent chromatin remodeling with DNA double-strand break repair. Cell 119: 777–788. 15607975 10.1016/j.cell.2004.11.033

[10] van Attikum H, Fritsch O, Gasser SM (2007) Distinct roles for SWR1 and INO80 chromatin remodeling complexes at chromosomal double-strand breaks. EMBO J 26: 4113–4125. 17762868 10.1038/sj.emboj.7601835 PMC2230671

[11] Wu S, Shi Y, Mulligan P, Gay F, Landry J, et al (2007) A YY1-INO80 complex regulates genomic stability through homologous recombination-based repair. Nat Struct Mol Biol 14: 1165–1172. 18026119 10.1038/nsmb1332 PMC2754171

[12] Park JH, Park EJ, Lee HS, Kim SJ, Hur SK, et al (2006) Mammalian SWI/SNF complexes facilitate DNA double-strand break repair by promoting gamma-H2AX induction. EMBO J 25: 3986–3997. 16932743 10.1038/sj.emboj.7601291 PMC1560357

[13] Doil C, Mailand N, Bekker-Jensen S, Menard P, Larsen DH, et al (2009) RNF168 binds and amplifies ubiquitin conjugates on damaged chromosomes to allow accumulation of repair proteins. Cell 136: 435–446. 19203579 10.1016/j.cell.2008.12.041

[14] Mailand N, Bekker-Jensen S, Faustrup H, Melander F, Bartek J, et al (2007) RNF8 ubiquitylates histones at DNA double-strand breaks and promotes assembly of repair proteins. Cell 131: 887–900. 18001824 10.1016/j.cell.2007.09.040

[15] Stewart GS, Panier S, Townsend K, Al-Hakim AK, Kolas NK, et al (2009) The RIDDLE syndrome protein mediates a ubiquitin-dependent signaling cascade at sites of DNA damage. Cell 136: 420–434. 19203578 10.1016/j.cell.2008.12.042

[16] Wang B, Elledge SJ (2007) Ubc13/Rnf8 ubiquitin ligases control foci formation of the Rap80/Abraxas/Brca1/Brcc36 complex in response to DNA damage. Proc Natl Acad Sci U S A 104: 20759–20763. 18077395 10.1073/pnas.0710061104 PMC2410075

[17] Goldberg M, Stucki M, Falck J, D’Amours D, Rahman D, et al (2003) MDC1 is required for the intra-S-phase DNA damage checkpoint. Nature 421: 952–956. 12607003 10.1038/nature01445

[18] Lou Z, Minter-Dykhouse K, Franco S, Gostissa M, Rivera MA, et al (2006) MDC1 maintains genomic stability by participating in the amplification of ATM-dependent DNA damage signals. Mol Cell 21: 187–200. 16427009 10.1016/j.molcel.2005.11.025

[19] Bekker-Jensen S, Mailand N (2010) Assembly and function of DNA double-strand break repair foci in mammalian cells. DNA Repair 9: 1219–1228. 21035408 10.1016/j.dnarep.2010.09.010

[20] Burma S, Chen BP, Murphy M, Kurimasa A, Chen DJ (2001) ATM phosphorylates histone H2AX in response to DNA double-strand breaks. J Biol Chem 276: 42462–42467. 11571274 10.1074/jbc.C100466200

[21] Kobayashi J, Tauchi H, Sakamoto S, Nakamura A, Morishima K, et al (2002) NBS1 localizes to gamma-H2AX foci through interaction with the FHA/BRCT domain. Curr Biol 12: 1846–1851. 12419185 10.1016/s0960-9822(02)01259-9

[22] Stewart GS, Wang B, Bignell CR, Taylor AM, Elledge SJ (2003) MDC1 is a mediator of the mammalian DNA damage checkpoint. Nature 421: 961–966. 12607005 10.1038/nature01446

[23] Celeste A, Petersen S, Romanienko PJ, Fernandez-Capetillo O, Chen HT, et al (2002) Genomic instability in mice lacking histone H2AX. Science 296: 922–927. 11934988 10.1126/science.1069398 PMC4721576

[24] Kobayashi J, Tauchi H, Chen B, Burma S, Tashiro S, et al (2009) Histone H2AX participates the DNA damage-induced ATM activation through interaction with NBS1. Biochem Biophys Res Commun 380: 752–757. 19338747 10.1016/j.bbrc.2009.01.109

[25] Mongelard F, Bouvet P (2007) Nucleolin: a multiFACeTed protein. Trends Cell Biol 17: 80–86. 17157503 10.1016/j.tcb.2006.11.010

[26] Kim K, Dimitrova DD, Carta KM, Saxena A, Daras M, et al (2005) Novel checkpoint response to genotoxic stress mediated by nucleolin-replication protein a complex formation. Mol Cell Biol 25: 2463–2474. 15743838 10.1128/MCB.25.6.2463-2474.2005 PMC1061594

[27] De A, Donahue SL, Tabah A, Castro NE, Mraz N, et al (2006) A novel interaction of nucleolin with Rad51. Biochem Biophys Res Commun 344: 206–213. 16600179 10.1016/j.bbrc.2006.03.113

[28] Takagi M, Absalon MJ, McLure KG, Kastan MB (2005) Regulation of p53 translation and induction after DNA damage by ribosomal protein L26 and nucleolin. Cell 123: 49–63. 16213212 10.1016/j.cell.2005.07.034

[29] Rogakou EP, Boon C, Redon C, Bonner WM (1999) Megabase chromatin domains involved in DNA double-strand breaks in vivo. J Cell Biol 146: 905–916. 10477747 10.1083/jcb.146.5.905 PMC2169482

[30] Kong X, Mohanty SK, Stephens J, Heale JT, Gomez-Godinez V, et al (2009) Comparative analysis of different laser systems to study cellular responses to DNA damage in mammalian cells. Nucleic Acids Res 37: e68. 19357094 10.1093/nar/gkp221 PMC2685111

[31] Yano K, Morotomi-Yano K, Adachi N, Akiyama H (2009) Molecular mechanism of protein assembly on DNA double-strand breaks in the non-homologous end-joining pathway. J Radiat Res (Tokyo) 50: 97–108. 19346677 10.1269/jrr.08119

[32] Berkovich E, Monnat RJ Jr, Kastan MB (2007) Roles of ATM and NBS1 in chromatin structure modulation and DNA double-strand break repair. Nat Cell Biol 9: 683–690. 17486112 10.1038/ncb1599

[33] Schultz LB, Chehab NH, Malikzay A, Halazonetis TD (2000) p53 binding protein 1 (53 BP1) is an early participant in the cellular response to DNA double-strand breaks. J Cell Biol 151: 1381–1390. 11134068 10.1083/jcb.151.7.1381 PMC2150674

[34] Yogev O, Saadon K, Anzi S, Inoue K, Shaulian E (2008) DNA damage-dependent translocation of B23 and p19 ARF is regulated by the Jun N-terminal kinase pathway. Cancer Res 68: 1398–1406. 18316603 10.1158/0008-5472.CAN-07-2865

[35] Ugrinova I, Monier K, Ivaldi C, Thiry M, Storck S, et al (2007) Inactivation of nucleolin leads to nucleolar disruption, cell cycle arrest and defects in centrosome duplication. BMC Mol Biol 8: e66. 10.1186/1471-2199-8-66 PMC1976620 17692122

[36] Pierce AJ, Jasin M (2005) Measuring recombination proficiency in mouse embryonic stem cells. Methods Mol Biol 291: 373–384. 15502236 10.1385/1-59259-840-4:373

[37] Mansour WY, Schumacher S, Rosskopf R, Rhein T, Schmidt-Petersen F, et al (2008) Hierarchy of nonhomologous end-joining, single-strand annealing and gene conversion at site-directed DNA double-strand breaks. Nucleic Acids Res 36: 4088–4098. 18539610 10.1093/nar/gkn347 PMC2475611

[38] Nakamura K, Kato A, Kobayashi J, Yanagihara H, Sakamoto S, et al (2011) Regulation of homologous recombination by RNF20-dependent H2B ubiquitination. Mol Cell 41: 515–528. 21362548 10.1016/j.molcel.2011.02.002

[39] Angelov D, Bondarenko VA, Almagro S, Menoni H, Mongelard F, et al (2006) Nucleolin is a histone chaperone with FACT-like activity and assists remodeling of nucleosomes. EMBO J 25: 1669–1679. 16601700 10.1038/sj.emboj.7601046 PMC1440837

[40] Tsukuda T, Fleming AB, Nickoloff JA, Osley MA (2005) Chromatin remodelling at a DNA double-strand break site in Saccharomyces cerevisiae. Nature 438: 379–383. 16292314 10.1038/nature04148 PMC1388271

[41] Murr R, Loizou JI, Yang YG, Cuenin C, Li H, et al (2006) Histone acetylation by Trrap-Tip60 modulates loading of repair proteins and repair of DNA double-strand breaks. Nat Cell Biol 8: 91–99. 16341205 10.1038/ncb1343

[42] Ogiwara H, Ui A, Otsuka A, Satoh H, Yokomi I, et al (2011) Histone acetylation by CBP and p300 at double-strand break sites facilitates SWI/SNF chromatin remodeling and the recruitment of non-homologous end joining factors. Oncogene 30: 2135–2146. 21217779 10.1038/onc.2010.592

[43] Lan L, Nakajima S, Hatakeyama K, Hoshi M, Watanabe R, et al (2010) The ACF1 complex is required for DNA double-strand break repair in human cells. Mol Cell 40: 976–987. 21172662 10.1016/j.molcel.2010.12.003

[44] Andersen JS, Lyon CE, Fox AH, Leung AK, Lam YM, et al (2002) Directed proteomic analysis of the human nucleolus. Curr Biol 12: 1–11. 11790298 10.1016/s0960-9822(01)00650-9

[45] Ganley AR, Ide S, Saka K, Kobayashi T (2009) The effect of replication initiation on gene amplification in the rDNA and its relationship to aging. Mol Cell 35: 683–693. 19748361 10.1016/j.molcel.2009.07.012

[46] Ide S, Miyazaki T, Maki H, Kobayashi T (2010) Abundance of ribosomal RNA gene copies maintains genome integrity. Science 327: 693–696. 20133573 10.1126/science.1179044

[47] Torres-Rosell J, Sunjevaric I, De Piccoli G, Sacher M, Eckert-Boulet N, et al (2007) The Smc5-Smc6 complex and SUMO modification of Rad52 regulates recombinational repair at the ribosomal gene locus. Nat Cell Biol 9: 923–931. 17643116 10.1038/ncb1619

[48] Farin K, Schokoroy S, Haklai R, Cohen-Or I, Elad-Sfadia G, et al (2011) Oncogenic Synergism between ErbB1, Nucleolin, and Mutant Ras. Cancer Res 71: 2140–2151. 21257709 10.1158/0008-5472.CAN-10-2887

[49] Kobayashi J, Okui M, Asaithamby A, Burma S, Chen BP, et al (2010) WRN participates in translesion synthesis pathway through interaction with NBS1. Mech Ageing Dev 131: 436–444. 20600238 10.1016/j.mad.2010.06.005 PMC2911442

