# Supplementary figures and images for: Nucleolin Participates in DNA Double-Strand Break-Induced Damage Response through MDC1-Dependent Pathway
Source: PLoS One. 2012 Nov 7;7(11):e49245. doi: 10.1371/journal.pone.0049245 (PMC3492271; doi:10.1371/journal.pone.0049245)

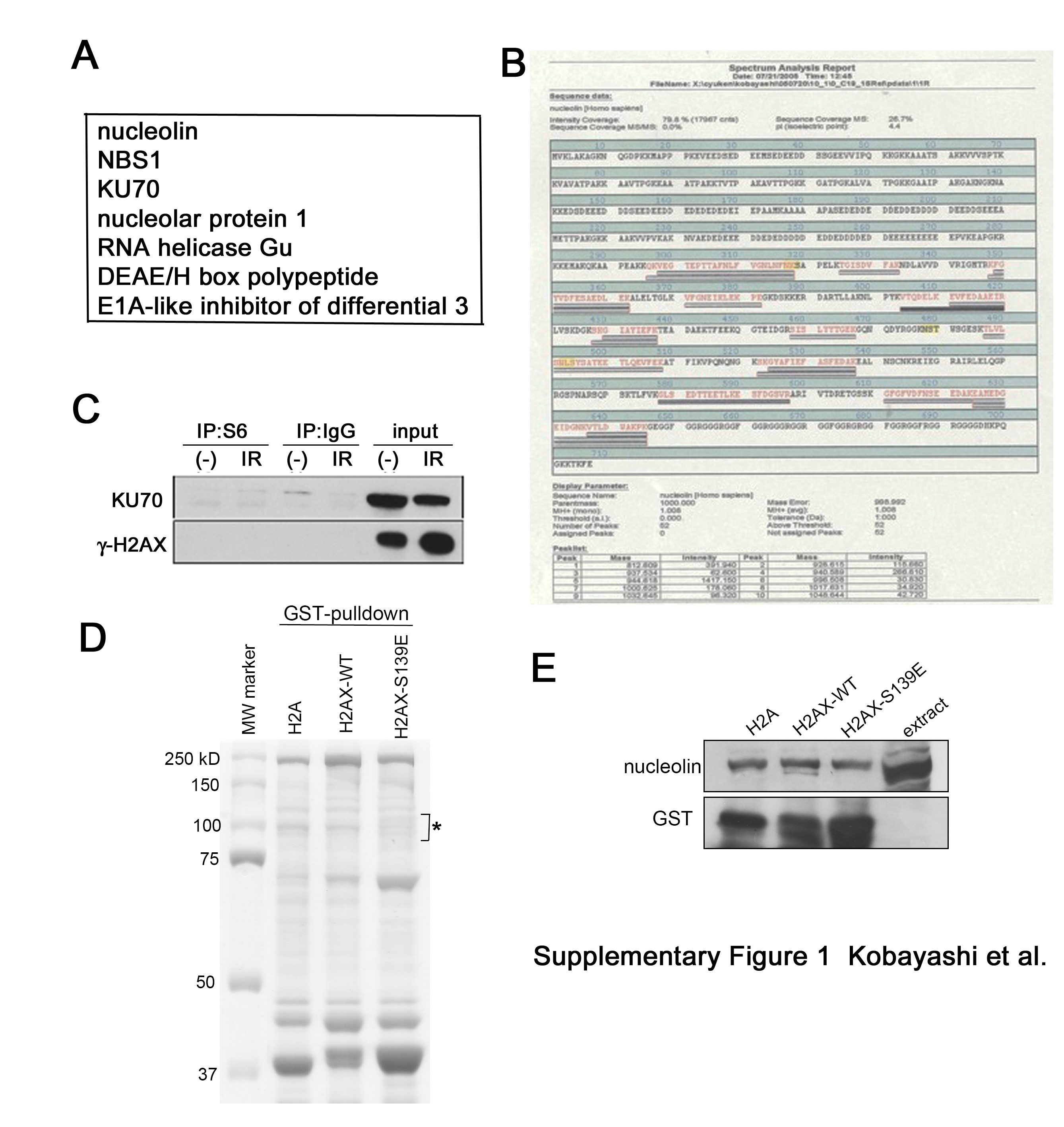

Supplement: Figure S1 — Identification of nucleolin as an associating protein with γ-H2AX. (A) List of H2AX-binding protein candidates by proteomics analysis. (B) The result of MASCOT analysis about the band identified as nucleolin. (C) Extracts from normal lymphoblastoid cells with or without IR (10 Gy) were immunoprecipitated with anti-ribosomal protein S6 antibody or normal rabbit IgG, and then the immuno-complexes were detected by Western blot analysis using indicated antibodies. (D)(E) Pulldowns by GST-H2A or GST H2AX were carried out from the nuclear extract of HeLa cells. Proteins were visualized by CBB staining (D). Precipitated nucleolin was visualized by Western blot (E). Extract: input nuclear extract only. (TIF) [file pone.0049245.s001.tif]

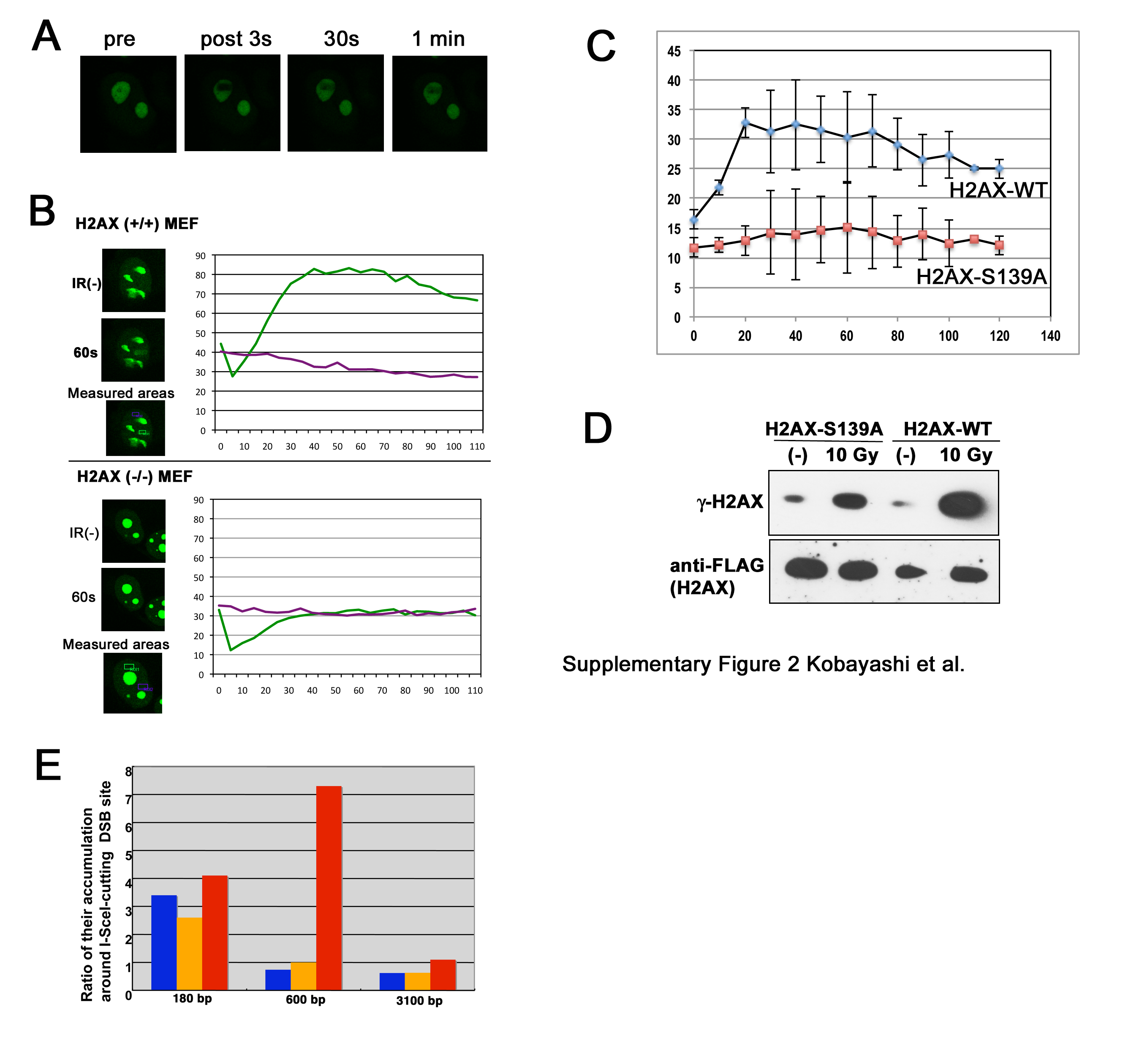

Supplement: Figure S2 — Nucleolin accumulates to DSB damage sites. (A) GFP-nucleolin did not accumulate in nucleolus following laser micro-irradiation in U2OS cells. (B) Laser micro-irradiation was performed in H2AX (+/+) or H2AX (−/−) mouse cells. Green line: fluorescence at micro-irradiated area, Purple line: fluorescence at un-irradiated sites. (C) H2AX (−/−) mouse cells were transfected by GFP-nucleolin and FLAG-H2AX (WT) or FLAG-H2AX (S139A), and after 2 days laser micro-irradiation was performed. (D) Expression of ectopic H2AX and its phosphorylation in (C) were confirmed by Western blot using anti-FLAG antibody and anti-γ-H2AX antibody. FLAG-H2AX (S139A)-expressing cells also showed its phosphorylation, suggesting that other SQ motifs such as serine 135) in H2AX may be phosphorylated in response to DSB damage. (E) Detection of nucleolin accumulation around DSB damage sites in MRC5SV by ChIP analysis. (TIF) [file pone.0049245.s002.tif]

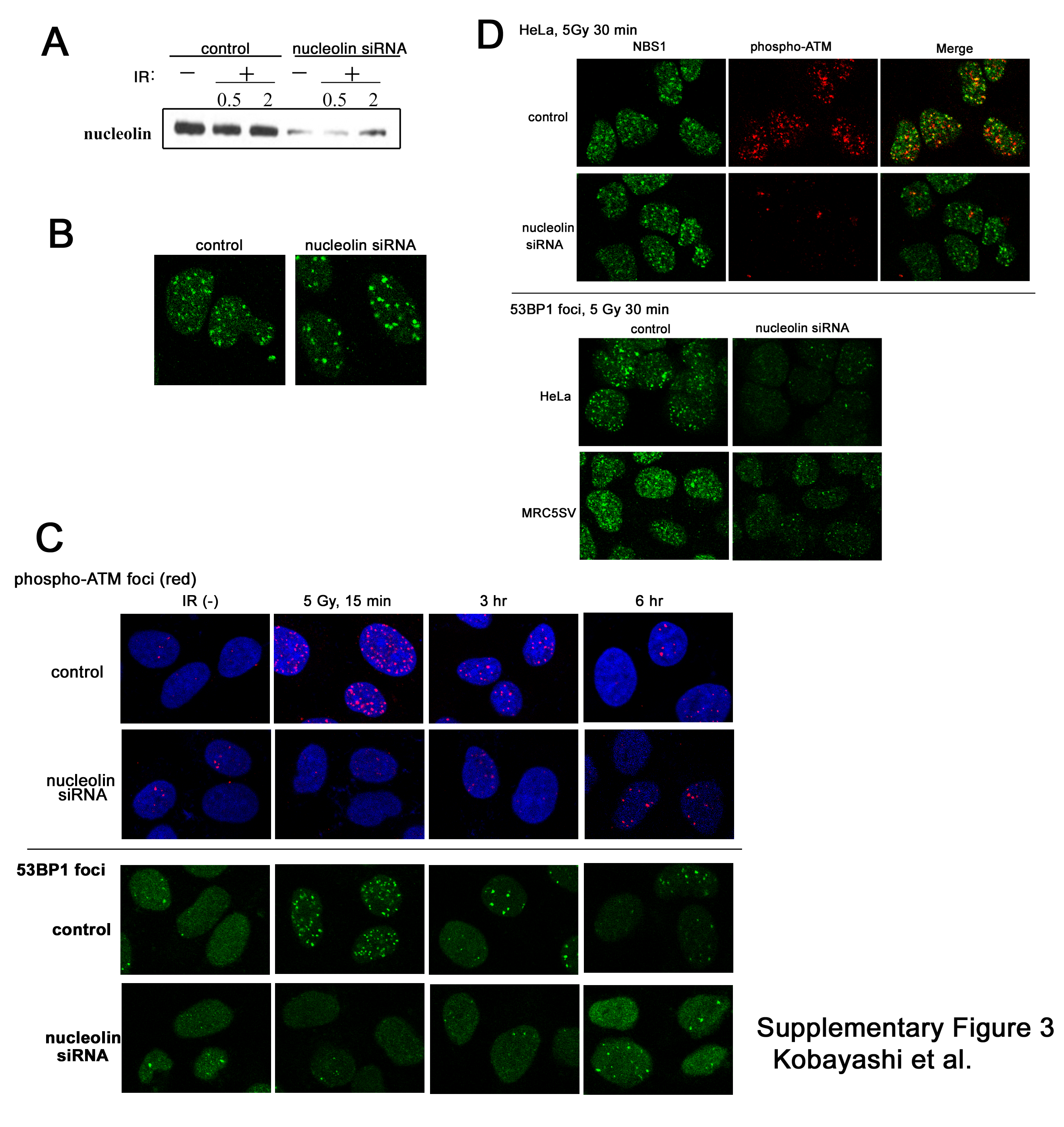

Supplement: Figure S3 — IR-induced focus formation of nucleolin-knockdown cells. (A) Our designing siRNA effectively reduced nucleolin protein in HeLa cells. (B)(C) U2OS cells were transfected by nucleolin siRNA or negative control siRNA, and after 2 days these cells were irradiated by 5 Gy of γ-ray. After 30 minutes, their cells were fixed and immuno-staining was performed using anti-MRE11 antibody (B) or indicated antibodies (C). phospho-ATM (red) or 53 BP1 (green) foci-positive cell were counted and these data are shown in Figure 3B. (D) Nucleolin-knockdown repressed the focus formation of phospho-ATM and 53 BP1. HeLa cells were transfected by nucleolin siRNA or negative control siRNA, and after 2 days these cells were irradiated by 5 Gy of γ-ray. After 30 minutes, their cells were fixed and immuno-staining was performed using indicated antibodies. (TIF) [file pone.0049245.s003.tif]

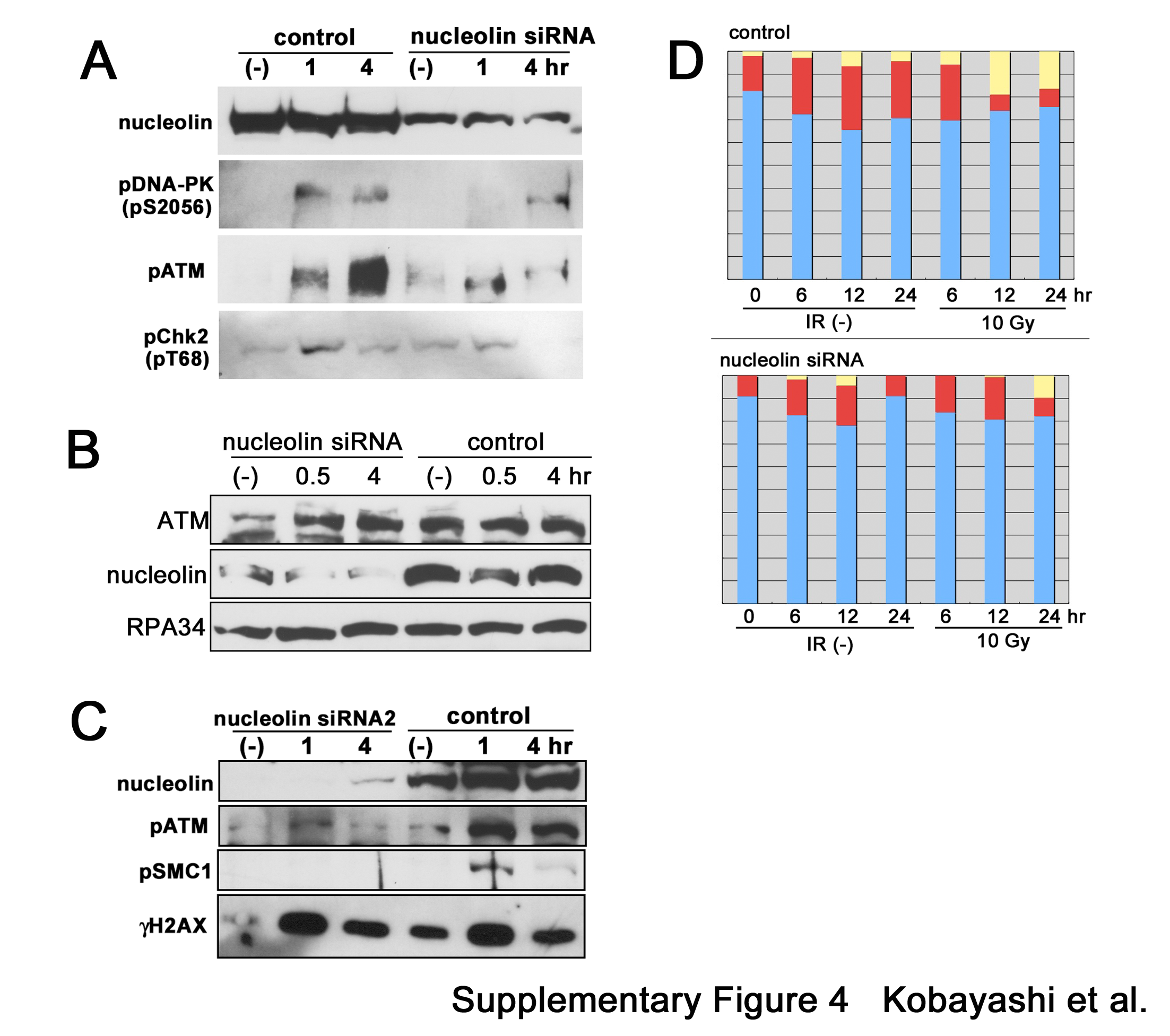

Supplement: Figure S4 — Nucleolin contributes to ATM-related pathway. MRC5SV cells (A) were transfected by nucleolin siRNA, while U2OS cells were transfected by nucleolin siRNA (B) or nucleolin siRNA2 (QIAGEN)(C). After 2 days, these cells were treated by 5 Gy of γ-ray and were harvested at indicated times after treatment, and analyzed by Western blot using indicated antibodies. (D) Nucleolin-knockdown abolished G2 checkpoint. 48BR cells were transfected by nucleolin siRNA. After 2 days, these cells were irradiated by 10 Gy of γ-ray and were fixed at indicated times by ethanol. After staining them by propidium iodide, the distribution of cell cycle was analyzed by flowcytometer. Blue column, G1 phase; red column, S phase; yellow column, G2/M phase cells. (TIF) [file pone.0049245.s004.tif]

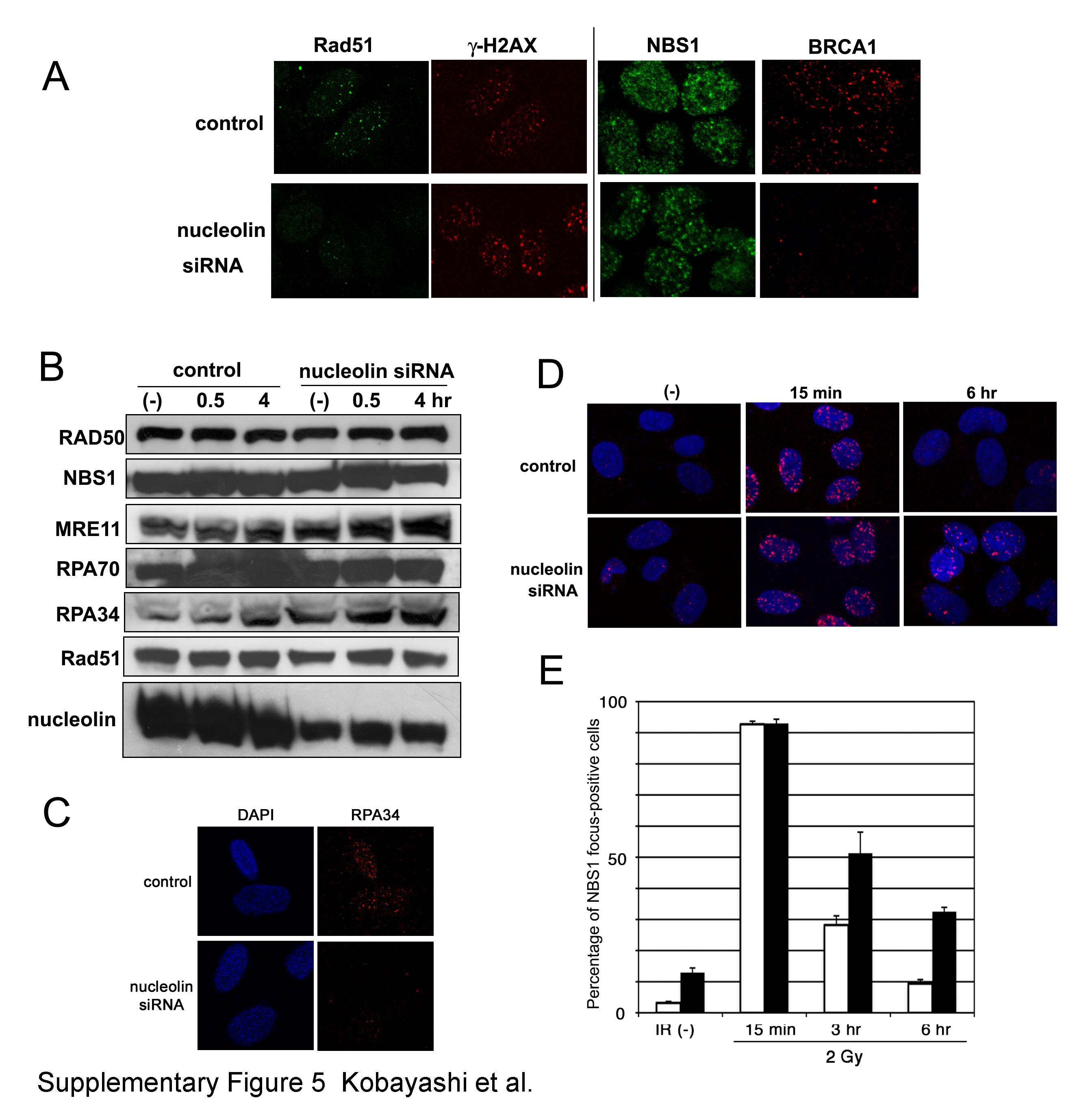

Supplement: Figure S5 — Nucleolin participates in DSB repair pathway. U2OS cells were transfected by nucleolin siRNA or negative control siRNA, and after 2 days these cells were irradiated by γ-ray. Their cells were fixed and immuno-staining was performed using anti-Rad51 and anti-BRCA1 (A), anti-RPA34(C), anti-γ-H2AX (D) or anti-NBS1 (E) antibodies. Percentage of focus-positive cells at indicated times after irradiation were counted under fluorescence microscope. Open column: control, closed column: nucleolin siRNA. (B) 48BR cells were transfected by nucleolin siRNA. After 2 days, these cells were irradiated by 5 Gy of γ-ray and were harvested at indicated times after IR and analyzed by Western blot using indicated antibodies. (TIF) [file pone.0049245.s005.tif]

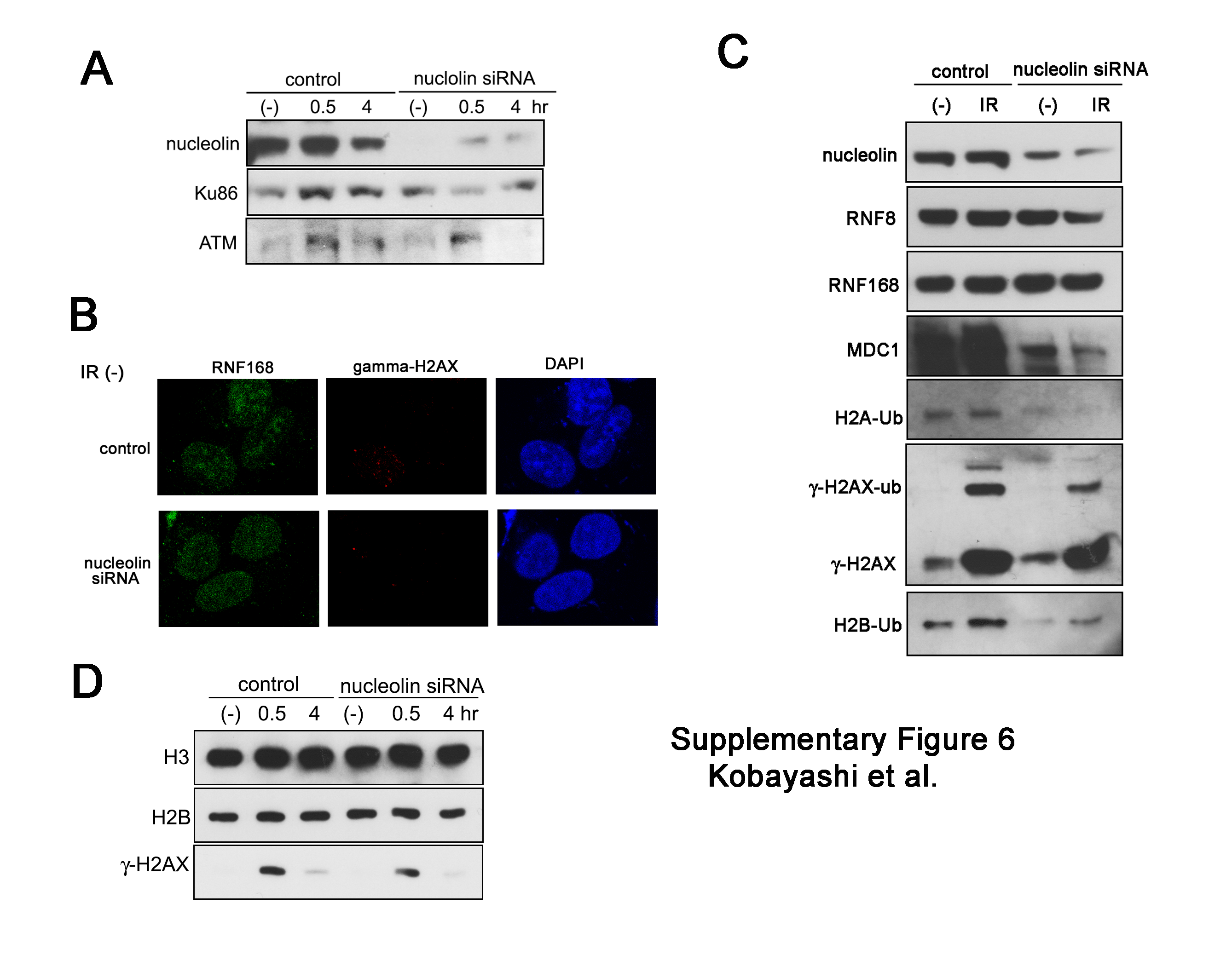

Supplement: Figure S6 — Nucleolin contributes to MDC1-dependent damage responses. (A) IR-induced accumulation of KU and ATM was abolished by repression of nucleolin. U2OS cells were transfected by nucleolin siRNA. After 2 days, these cells were irradiated by 10 Gy of γ-ray and were harvested at indicated times after IR. After preparation of nucleoplasm (nuclear supernatant) and chromatin extracts, chromatin association of KU86 and ATM was analyzed by Western blot. (B) U2OS cells were transfected by nucleolin siRNA or negative control siRNA, and after 2 days these cells (without irradiation) were immuno-stained using anti-RNF168 antibody. (C)(D) U2OS cells were transfected by nucleolin siRNA. After 2 days, these cells were irradiated by 10 Gy of γ-ray and were harvested at indicated times after IR. After preparation of chromatin extracts, chromatin associated proteins were analyzed by Western blot using indicated antibodies. Ubiquitination of H2AX was estimated with its molecular weight using anti-γ-H2AX antibody. (TIF) [file pone.0049245.s006.tif]

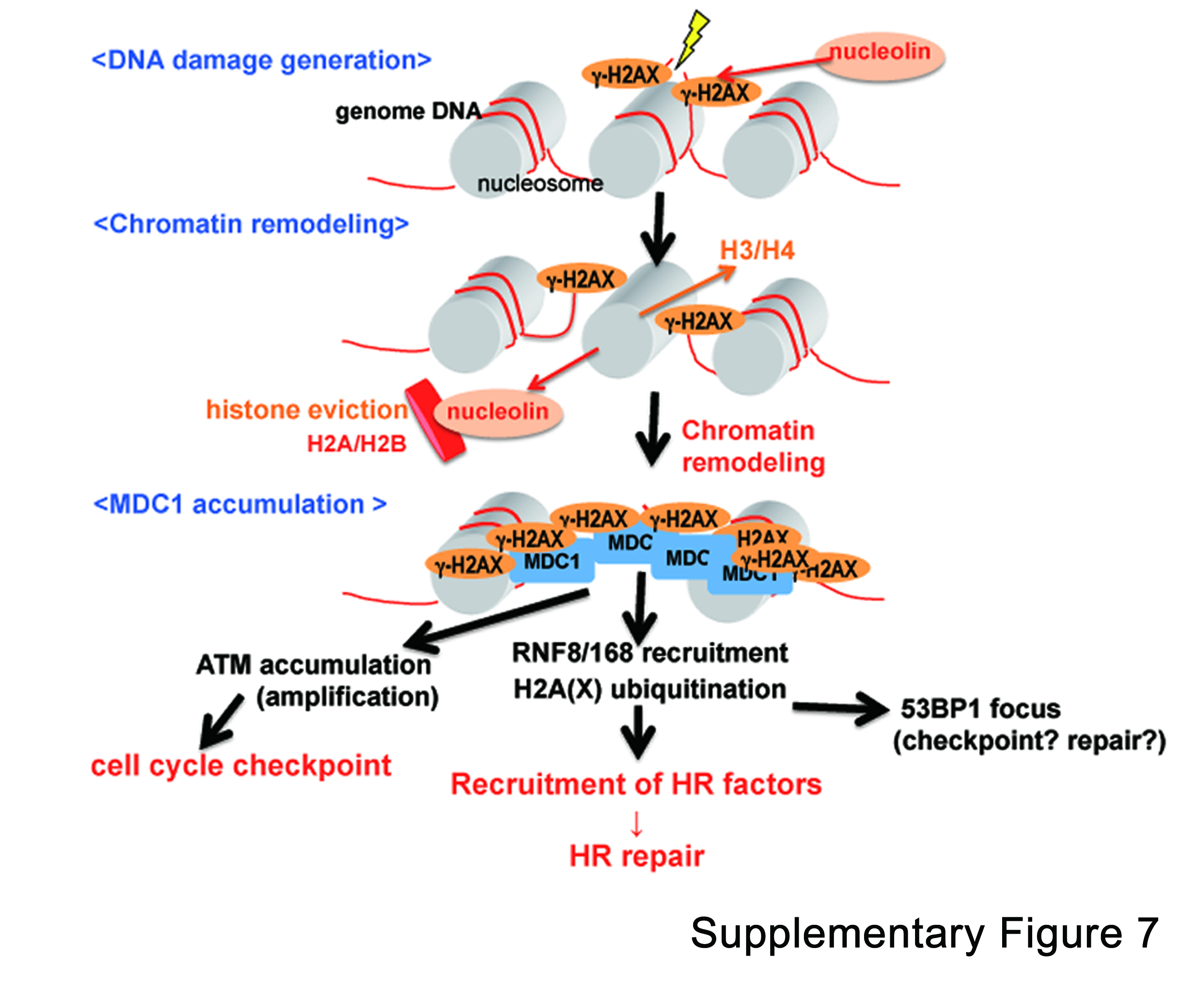

Supplement: Figure S7 — Nucleolin participates into MDC1-related DNA damage responses through histone eviction. Nucleolin recruits to DSB damage sites in H2AX-dependent manner and then promotes histone eviction and subsequent histone remodeling through binding with histone H2A/H2B. This histone eviction and remodeling facilitates chromatin association of MDC1 at DSB sites. As a result, MDC1-related DNA damage responses, such as ATM-dependent checkpoint and HR repair, are initiated. (TIF) [file pone.0049245.s007.tif]
